# Assessing the causal relationship between COVID-19 and post-COVID-19 syndrome: A Mendelian randomisation study

**DOI:** 10.7189/jogh.13.06054

**Published:** 2023-12-13

**Authors:** Yiming Tao, Rui Zhao, Jie Han, Yongsheng Li

**Affiliations:** 1 Department of Intensive Care Medicine, Tongji Hospital, Tongji Medical College, Huazhong University of Science and Technology, Hankou, Wuhan, China; 2 Department of Laboratory Medicine, The First Affiliated Hospital of Chongqing Medical University, Chongqing, China; 3 Department of Emergency, Qingdao Municipal Hospital, School of Medicine, Qingdao University, Qingdao, China

## Abstract

**Background:**

In the aftermath of the coronavirus disease 2019 (COVID-19) pandemic, we sought to explore the causal association between COVID-19 and 17 prevalent post-COVID-19 syndrome (PCS) symptoms using Mendelian randomisation (MR) methodology.

**Methods:**

We used 22 extensive genome-wide association study (GWAS) data sets, incorporating genetic variants as instrumental variables. Univariate Mendelian randomisation (UVMR) analyses involved 15 single nucleotide polymorphisms (SNPs) for COVID-19 patients, 33 for hospitalised COVID-19 patients, and 29 for patients with severe respiratory symptoms due to COVID-19. Furthermore, we further used multivariable Mendelian randomisation (MVMR) analyses based on 93 SNPs for COVID-19 patients, 105 for hospitalised COVID-19 patients, and 99 for patients with severe respiratory symptoms due to COVID-19. With these analyses, we aimed to assess the causal associations between varying levels of COVID-19 infection and 17 prevalent PCS symptoms while accounting for the influence of educational and income levels.

**Results:**

UVMR analysis identified potential causal effects of COVID-19 genetic susceptibility on myalgia and pain in various regions. Hospitalised COVID-19 was potentially linked to erectile dysfunction and alopecia areata. Very severe respiratory confirmed patients exhibited increased pain and tobacco use. Meanwhile, the MVMR analysis demonstrated a potential causal link between hospitalised COVID-19 and heart arrhythmia, and a protective effect of COVID-19 on tobacco use after adjusting for educational and income levels.

**Conclusions:**

Our MR analysis provides compelling evidence of causal associations between genetic susceptibility to COVID-19 and specific PCS symptoms, in which educational and income levels play a mediating role. These findings shed light on PCS pathogenesis and underscore the importance of considering social factors in its management. Tailored interventions and policies are crucial for PCS-affected individuals' well-being. Further research is needed to explore the impact of social determinants on COVID-19 patients and the wider population.

The coronavirus disease 2019 (COVID-19) pandemic has had significant consequences beyond its acute phase, with post-COVID-19 Syndrome (PCS) emerging as a significant concern. Approximately 10-30% of COVID-19 survivors may develop PCS, leading to substantial medical and socioeconomic challenges [1,2]. However, its underlying cause remains uncertain, characterized by complex interactions between viral toxicity and socio-psychological factors [3,4]. With this gap in mind, we sought to better understand the relationship between COVID-19 infection and PCS, elucidating the specific PCS symptoms influenced by varying degrees of COVID-19 severity. Our aim was to pinpoint the key contributors to PCS development and thus provide insights into effective prevention and management strategies.

Previous investigations into PCS have primarily relied on observational studies and narrative reviews. However, the complex and multifaceted nature of PCS, as well as the diversity of treatment interventions required, has hindered the execution of randomised controlled trials (RCTs) [1]. Observational studies, while valuable, are limited by their inability to manipulate participant grouping and treatment interventions, rendering them susceptible to biases and confounding variables [5]. Additionally, the distinction between COVID-19 infection and other factors contributing to similar symptoms further impedes establishment of a causal link between COVID-19 and PCS [6]. To overcome these limitations and try to determine causal inference, we used Mendelian randomisation (MR) analysis, which utilises naturally occurring genetic variations as “instrumental variables” to mimic the effects of RCTs [7], allowing us to explore the causal impacts of COVID-19 infection on various PCS symptoms without subjecting participants to COVID-19 exposure, thereby addressing ethical concerns [8]. Furthermore, existing MR studies on PCS only consider the association between COVID-19 and a specific subtype of PCS or fail to consider the influence of varying degrees of COVID-19 infection severity. Consequently, to comprehensively comprehend the causal connection between COVID-19 and PCS, it is imperative to conduct more comprehensive and stratified Mendelian randomisation studies [9-11]. Here we used the two-sample univariable Mendelian randomisation (UVMR) approach to evaluate the causal effects of COVID-19, COVID-19-related hospitalisation, and COVID-19 with severe respiratory symptoms on 17 prevalent PCS symptoms, while accounting for social factors such as education level and income level [12], which have been associated with PCS susceptibility. These social factors may indirectly influence PCS occurrence and severity through their impact on psychological well-being and vaccination rates [13-16]. To mitigate potential confounding, we incorporated education level and income level into our analysis using multivariable Mendelian randomisation (MVMR). With this comprehensive research design, we sought to more deeply understand the potential repercussions of COVID-19 infection on PCS, while considering the moderating effects of social factors. By meticulously assessing the causal association between COVID-19 infection and PCS and effectively addressing potential confounding variables, we aimed to offer valuable insights into the understanding and management of PCS.

## METHODS

### Study design

We performed a two-sample MR analysis based on data from 22 extensive genome-wide association study (GWAS) datasets as our primary sources for both exposure and outcome data. Our primary objective was to explore the causal relationship between COVID-19 infection and PCS, with a specific focus on 17 prevalent PCS-related symptoms. We based our selection of these symptoms on comprehensive reviews of high-quality literature [1-4], supplemented by insights from the authors' clinical expertise.

**Figure 1:**
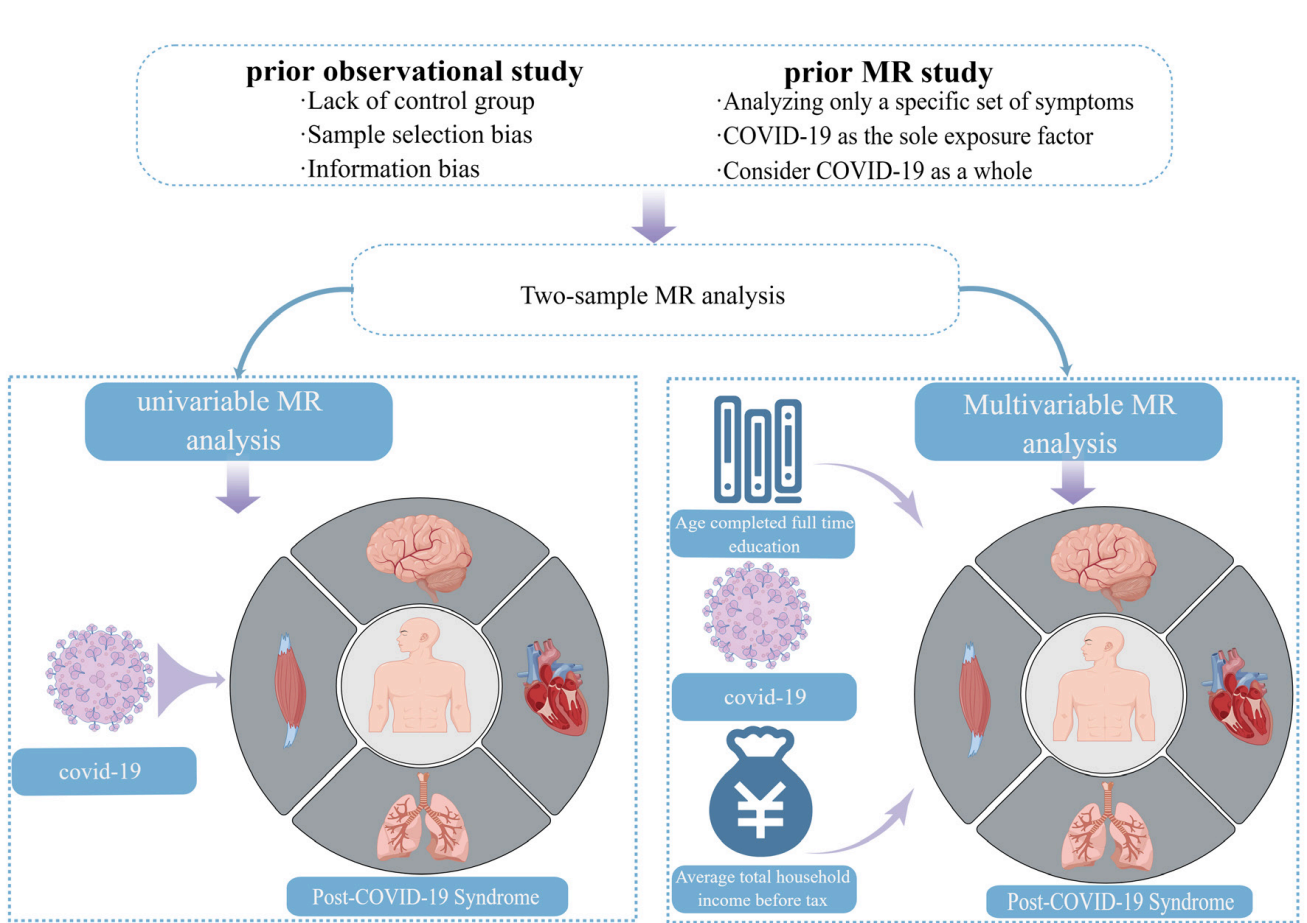
Workflow of Mendelian randomization study revealing causality from Covid-19 on post-COVID-19 syndrome.

To address potential confounding factors, we applied the MVRM methodology, including educational and income levels as covariates (**Figure 1**). This analysis relies on three core assumptions: first, it assumes a correlation between genetic variation and the exposure factor; second, it posits that genetic variation remains unaffected by any confounding factors related to the outcome; and lastly, it suggests that the genetic variation's influence on the outcome is exclusively mediated through the exposure factor, excluding any other confounding factors [17].

Furthermore, we conducted a meta-analysis on the results of the univariable MR analyses, sourcing the outcome data from Finngen, the UK Biobank, and the European Bioinformatics Institute (EBI), which provided additional validation for the findings derived from the univariable MR analyses. We performed statistical analyses using the “TwoSampleMR” package (version 0.5.6) and the “MendelianRandomization” package (version 0.7.0) in R, version 4.2.3 (R Core Team, Vienna, Austria).

### Data sets

#### GWAS data for exposure

We used publicly accessible GWAS summary statistics as primary data sources, focusing mainly on individuals of European descent to mitigate potential ethnic disparities. We collected GWAS data for COVID-19 patients, hospitalized COVID-19 patients, and patients with severe respiratory symptoms due to COVID-19, along with corresponding population controls, from the COVID-19 Host Genetics Initiative and other sources [18]. Additionally, we retrievd GWAS data on educational level and income level from the UK Biobank.

#### GWAS data for outcome

We identified a comprehensive range of 17 prevalent symptoms that serve as the primary outcome for PCS; they encompassed various manifestations, including malaise and fatigue, exertional dyspnoea, cardiac arrhythmia, disordered eating patterns, myalgia, dorsalgia, arthralgia, pain in various regions (limbs, back, neck, head, abdomen), sleeplessness or insomnia, memory loss, depressive symptoms, anxiety disorders, a history of suicide attempts, alcohol consumption, tobacco usage, erectile dysfunction, and alopecia areata. To prevent sample duplication, we obtained GWAS data from the FinnGen database, while we retrieved only shortness of breath data from the UK Biobank, enabling single-sample MR (Table S1 in the **Online Supplementary Document**).

#### Selection of genetic instruments

We employed a stringent criterion of *P* < 5 × 10^-8^ to select SNPs strongly associated with the exposure factor. We conducted clustering using a window size of 10 000 kb and an R^2^ < 0.001 threshold. To address potential bias, we harmonised the exposure and outcome variables, ensuring consistent matching of effect alleles within the same allele [19]. For each instrumental variable (IV), we systematically searched the PhenoScanner GWAS database, excluding any SNPs correlated with confounding factors. To assess the strength of the IVs, we computed F-statistics for all SNPs using the formula F = β^2^ exposure/SE^2^ exposure, ensuring F-values exceeded 10 to address biases from weak instrumental variables [20].

In the MVMR analysis, meeting the condition of F > 10 simultaneously for SNPs in all three exposure GWAS data sets was challenging. Hence, we used a fixed-effects model to calculate the F-statistic for the entire GWAS data set to assess the overall association between the IV and exposure.

### Statistical analysis

The primary approach for the two-sample MR analysis was the random-effects inverse-variance weighted (IVW) method, which effectively addresses heterogeneity among genetic instruments and improves the accuracy of estimates [21]. We also employed various other MR models such as weighted median, MR-Egger, simple mode, and weighted mode to validate causal associations [22]. Statistical significance was determined using a Bonferroni-corrected threshold of *P* < 0.00294 (*P* = 0.05/17), while *P*-values ranging from 0.00294 to 0.05 were considered indicative of suggestive evidence for a causal relationship [23]. Sensitivity analysis involved assessing heterogeneity, detecting and correcting outliers and pleiotropy using MR-pleiotropy residual sum and outlier (MR-PRESSO) and MR-Egger intercept test [24,25]. For each SNP, we conducted a leave-one-out analysis and generated a forest plot to evaluate their individual contributions.

To adjust for potential confounding effects, we performed MVMR analysis while incorporating educational level and income level. The multivariable IVW, multivariable MR-Egger, and multivariable MR-Lasso approaches were utilized [7]. The use of the multivariable IVW method yielded an all-encompassing estimation, while the implementation of the multivariable MR-Egger approach effectively addressed the issue of pleiotropy. Additionally, the incorporation of the multivariable MR-Lasso technique facilitated the selection of pertinent exposure variables [26], thereby bolstering the credibility and resilience of causal inference.

## RESULTS

In the UVMR analysis, 15 SNPs (F-values = 30.608-413.143) were extracted from GWAS data of COVID-19 patients after removing palindromic SNPs. Hospitalised COVID patients had 33 selected SNPs (F-values = 29.790-787.893) while very severe respiratory confirmed patients had 29 (F-values = 30.357-844.590). Subsequently, some SNPs were excluded based on outcome factors and outliers (Table S2 in the **Online Supplementary Document**).

We extracted 91 SNPs (F-value = 11.923) in the MVMR analysis, considering age, completed full-time education, average total household income before tax, and COVID-19 patients. Combining hospitalised COVID with exposure factors resulted in 102 SNPs, while combining very severe respiratory confirmed patients with COVID-19 yielded 96 (F-values = 15.888-15.114, respectively). Certain SNPs associated with 17 outcomes were removed based on second phenotypes and outliers (Table S3 in the **Online Supplementary Document**).

### UVMR analysis

**Figure 2:**
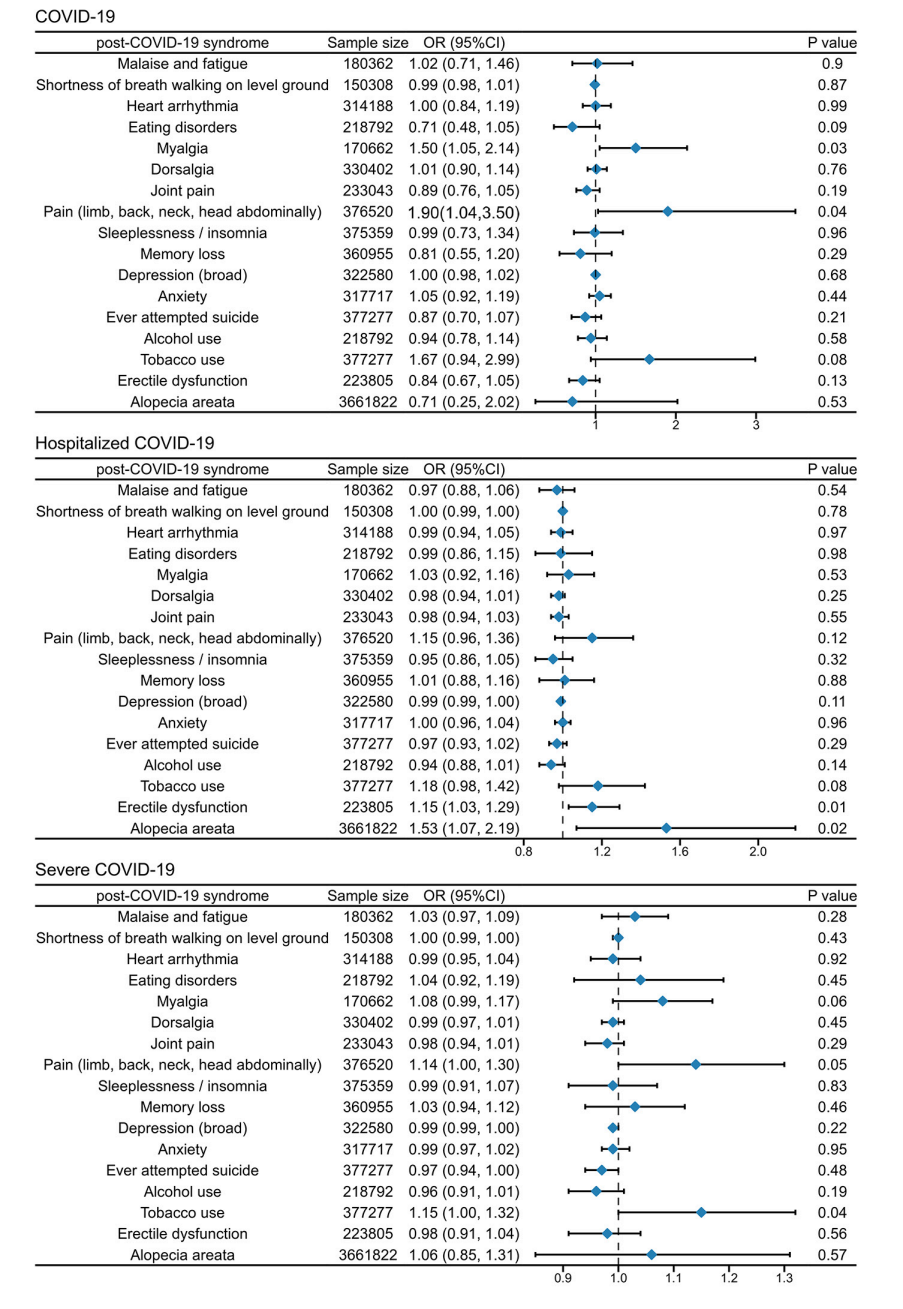
Forest plot for the univariate mendelian randomization. OR – odds ratio, CI – confidence interval.

The UVMR analysis suggested potentially harmful causal effects of COVID-19 genetic susceptibility on myalgia (IVW odds ratio (OR) = 1.5; 95% confidence interval (CI) = 1.05-2.14, *P* < 0.05) and pain in various body regions (IVW OR = 1.90; 95% CI = 1.04-3.50, *P* < 0.05). In the analysis of COVID-19 hospitalised patients as the exposure factor with erectile dysfunction and alopecia areata as the outcome factors, heterogeneity and its causes were identified (erectile dysfunction-rs67959919, alopecia areata-rs78314212). Following the removal of these outliers, we determined that COVID-19 hospitalised patients may experience a potentially detrimental impact on erectile dysfunction (IVW OR = 1.15; 95% CI = 1.03-1.29, *P* < 0.05) and alopecia areata (IVW OR = 1.531; 95% CI = 1.070-2.191, *P* = 0.02). For very severe respiratory confirmed patients with COVID-19, we found potentially harmful causal effects on pain in various body regions (IVW OR = 1.14; 95% CI = 1.00-1.30, *P* < 0.05) and tobacco use (IVW OR = 1.15; 95% CI = 1.00-1.32, *P* < 0.05). Sensitivity analysis did not reveal heterogeneity or pleiotropy, while the leave-one-out analysis also confirmed that individual SNPs did not affect the IVW estimates (**Figure 2**, Table S4 and Figures S1.1-S17.3 in the **Online Supplementary Document**). The results of the meta-analysis further supported the causal relationships between COVID-19 and myalgia, hospitalised COVID-19 and erectile dysfunction, as well as severe respiratory symptoms due to COVID-19 and widespread multi-site pain. However, due to potential issues related to sample overlap, our primary emphasis remains on the results of the original analyses (Figure 18 in the **Online Supplementary Document**).

### MVMR analysis

**Figure 3:**
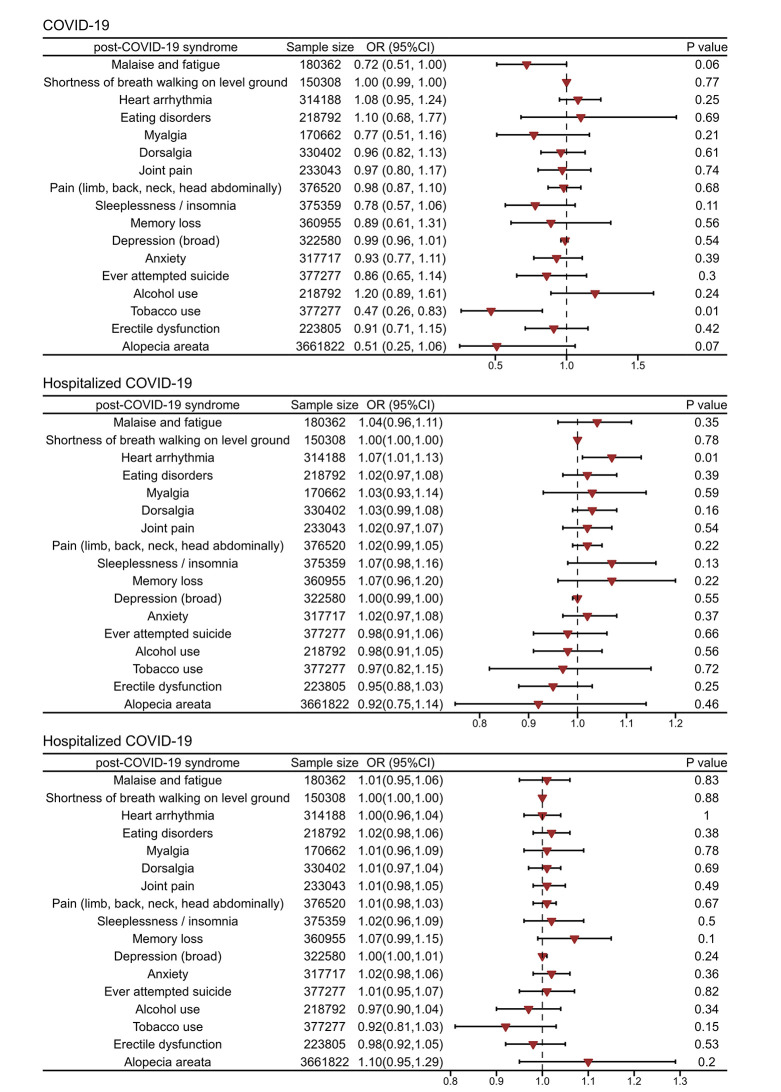
Forest plot for the multivariate mendelian randomization accounting for age completed full time education and average total household income before tax. OR – odds ratio, CI – confidence interval.

In the MVMR analysis, after adjusting for educational and income levels, the gene prediction findings suggest that hospitalisation with COVID-19 may be associated with a potentially detrimental causal effect on heart arrhythmia (MVIVW OR = 1.07; 95% CI = 1.01-1.133, *P* < 0.05). Notably, the MVMR analysis also showed a causal protective effect of COVID-19 susceptibility on tobacco use, potentially leading to a decrease in its prevalence (MVIVW OR = 0.47; 95% CI = 0.26-0.83, *P* < 0.05). This implies that individuals who possess a genetic predisposition to COVID-19 susceptibility may exhibit a diminished likelihood of engaging in tobacco use. The analysis showed no significant heterogeneity or pleiotropy, reinforcing the reliability of the findings (**Figure 3** and Table S5 in the **Online Supplementary Document**).

## DISCUSSION

Our primary finding is the establishment of a causal association between COVID-19 infection and PCS. This has significant implications for a wide population, with PCS's long-term impact surpassing initial estimates. While the exact aeitology of PCS remains unknown, we found that the severity of COVID-19 infection may contribute to its onset and progression through immune system effects, viral persistence, and inflammation [4]. Moreover, social determinants like education and income levels might influence PCS risk and severity [12]. Although RCTs specifically targeting PCS are scarce, we overcame these limitations by using a two-sample MR methodology to examine the causal association between varying degrees of COVID-19 infection and 17 prevalent symptoms of PCS. We observed that the genetic inclination toward susceptibility to COVID-19 might be linked to an elevated likelihood of experiencing myalgia, as well as pain in the limbs, back, neck, head, and abdomen. We also observed a correlation between the genetic predisposition to being hospitalised from COVID-19 and an escalated susceptibility to erectile dysfunction and alopecia areata. Moreover, individuals with a genetic predisposition to severe respiratory manifestations of COVID-19 may exhibit an augmented likelihood of experiencing pain in various regions of the body and engaging in tobacco use. These findings not only validate but also extend the outcomes of conventional observational studies exploring PCS.

Musculoskeletal symptoms, such as myalgia and headache, have been extensively documented in individuals who have recovered from COVID-19, with a notable proportion (up to 45.1%) experiencing persistent pain even after an eight-month period of recuperation [27]. This enduring pain may be attributed to prolonged inflammatory responses elicited by SARS-CoV-2, which involve abnormal mast cell reactions and heightened expression of ACE2 receptors [28]. Furthermore, factors such as posttraumatic stress disorder and anxiety may also contribute to the presence of these symptoms [27]. Our investigation provides further evidence supporting the causal relationship between COVID-19 and the occurrence of myalgia and pain in various regions of the body.

The association between COVID-19 and erectile dysfunction has been firmly established, as systemic inflammation and vascular impairment in the penile region are potential underlying factors [29]. Additionally, COVID-19's impact on the nervous system and psychological elements such as anxiety and depression may also contribute to sexual dysfunction [30]. Meanwhile, research on alopecia areata has been inconsistent. While certain studies have reported a rise in cases during the pandemic [31], others have found no direct correlation [32]. A recent meta-analysis has concluded that COVID-19 may worsen existing alopecia areata, but there is insufficient evidence to support its role in new cases [33]. The potential variation in findings can potentially be accounted for by disparities in mitigation strategies and psychological burdens across different countries. Our MR analysis establishes a connection between genetic susceptibility to being hospitalised from COVID-19 and the occurrence of erectile dysfunction and alopecia areata. This observed correlation can be attributed to the amplified psychological and economic challenges experienced by individuals who have been hospitalised with COVID-19, potentially augmenting their vulnerability to developing erectile dysfunction and alopecia areata.

In contrast, the relationship between COVID-19 infection and tobacco usage is intricate, with conflicting findings reported in various studies due to multiple confounding variables. A study conducted in Finland indicated a noticeable decrease in daily smoking rates from 2018 to 2020, suggesting that the COVID-19 pandemic did not substantially impact the ongoing decline in smoking prevalence [34]. Conversely, research from the Czech Republic showed that the psychological well-being of the population deteriorated during the pandemic, resulting in an increase in tobacco consumption [35]. Moreover, a cohort study in France found a significant association between smoking prevalence during the pandemic and educational attainment, indicating the influence of widening social inequality. Individuals with higher levels of education were more inclined to reduce tobacco consumption, while those with lower educational backgrounds were more prone to smoking [36]. Our MR analysis suggests that a genetic predisposition to severe COVID-19 in patients with confirmed respiratory symptoms may contribute to an elevated susceptibility to tobacco use.

We proceeded with additional MVMR analysis to examine the correlation between social and psychological factors and PCS and found that education and income acted as mediators in the relationship between COVID-19 and most PCS symptoms. Following adjustments, the causal connections between COVID-19 and symptoms such as myalgia, pain in multiple regions, erectile dysfunction, and alopecia areata were attenuated. Nevertheless, we did observe a causal association between genetic predisposition for hospitalized COVID-19 and arrhythmia. Multiple studies have provided evidence suggesting that SARS-CoV-2 possesses the capability to bind to ACE2 receptors located on cardiovascular cells [37]. Additionally, the inflammatory response triggered by the activation of macrophages and CD4+ T cells can directly hinder the normal functioning of endothelial cells and cardiomyocytes, consequently disrupting both microvascular and macrovascular endothelium [38]. This disruption ultimately culminates in the manifestation of arrhythmias.

Through MVMR analysis, we established a causal association between genetic susceptibility to COVID-19 and a diminished propensity for tobacco use. Conversely, the UVMR analysis indicated that those predisposed to severe respiratory symptoms had a higher risk of tobacco use. Socioeconomic factors, like education and income, significantly influenced tobacco usage among COVID-19 patients. Further research should comprehensively analyse the effects of PSC, education, income, and national policies on tobacco usage, while also exploring the underlying mechanisms involved.

In using a two-sample MR design to examine the association between three genetic predispositions for COVID-19 and 17 PCS symptoms and adhering to the assumptions inherent to the methodology, we aimed to overcome challenges such as residual confounding and reverse causation commonly encountered in observational studies. We identified strongly associated SNPs for COVID-19 genetic predisposition (*P* < 5 × 10^-8^). We excluded outliers and then performed UVMR analysis after excluding the second phenotype. Overall, our findings are suggestive of a causal relationships between COVID-19 infections and various PCS symptoms.

A notable strength of our study is the application of MVMR, which considers education and income as mediators influencing PCS through psychological well-being and behavioural interventions [14]. The varied repercussions of the COVID-19 pandemic on the economy, education, and mental well-being complicate the relationship between education, income, and PCS [12,13,39] (Figure S19 in the **Online Supplementary Document**). The use of MVMR proved to be effective in mitigating the influence of confounding variables, specifically education and income levels; our analysis thus highlights the impact of education and income on the development of PCS. Additional research is needed to understand the underlying mechanisms behind these associations.

We also acknowledge certain limitations in our study. First, while we used phenoscanner to exclude SNPs with known biological effects on the outcomes, a complete understanding of all SNPs’ functionality remains lacking, making it challenging to fully rule out pleiotropy. However, the effect estimates remained robust across different MR models and sensitivity analyses did not show significant pleiotropy. Second, the *P*-values from our MR models did not reach a threshold <0.00294. *P*-values falling between 0.00294 and 0.05 suggest potential associations. but may be susceptible to Type I errors. Applying Bonferroni correction could reduce statistical power, making it harder to identify causal links between COVID-19 and PCS. Lastly, the classification of COVID-19 phenotypes in our study based on disease severity and hospitalisation status lacked specific details on organ involvement, limiting a comprehensive evaluation of COVID-19's varied effects on PCS across different organ systems. Therefore, larger sample sizes are needed for more precise and reliable conclusions [40].

## CONCLUSION

In our MR analysis, we found evidence for the causal influence of genetic predisposition to varying degrees of COVID-19 infection on the heightened susceptibility to certain prevalent PCS. It is possible that several of these symptoms are potentially influenced by factors associated with educational attainment and income level, adding to a novel understanding of PCS pathogenesis and emphasising the significance of incorporating social and psychological determinants, alongside policy modifications, in the comprehensive approach to PCS treatment and management throughout and following the pandemic. Besides prioritising rehabilitation measures and pharmaceutical interventions, it is crucial to conduct further research on the impact of social and psychological factors and policy modifications on both COVID-19 patients and the broader population. Our study could inform the development of tailored interventions and holistic strategies aimed at improving the well-being of individuals affected by PCS.

## Additional material


Online Supplementary Document
