# Supplementary material for: Assessing the causal relationship between COVID-19 and post-COVID-19 syndrome: A Mendelian randomisation study
Source: J Glob Health. 2023 Dec 13;13:06054. doi: 10.7189/jogh.13.06054 (PMC10715454; doi:10.7189/jogh.13.06054)
Supplement: Online Supplementary Document [file jogh-13-06054-s001.zip › jogh-13-06054-s001.pdf]

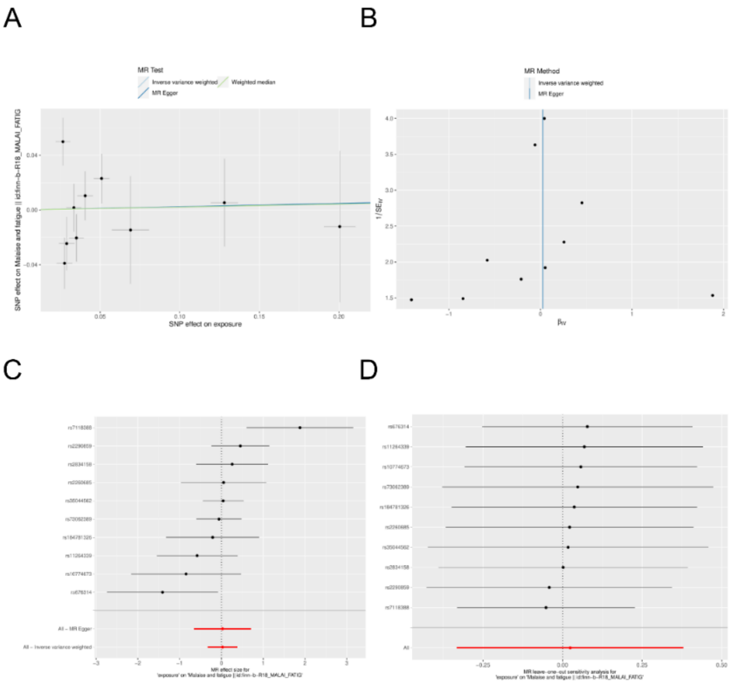

Figure.S1.1 Results of univariate Mendelian randomization of COVID-19-Malaise and fatigue.
（A. scatter plots of causality, B. funnel plots, C.forest plots of each SNPs, D.leave-one-out plots）

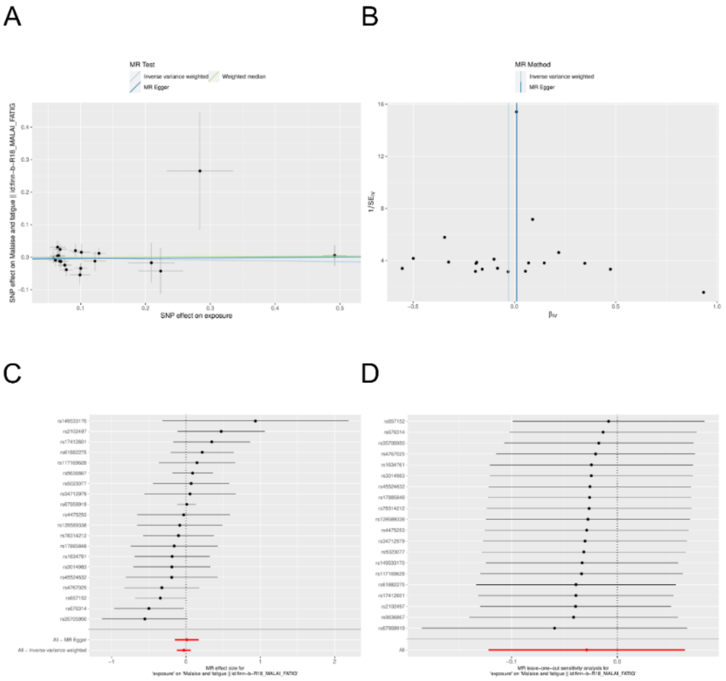

Figure.S1.2 Results of univariate Mendelian randomization of Hospitalized covid -Malaise and fatigue.
（A. scatter plots of causality, B. funnel plots, C.forest plots of each SNPs, D.leave-one-out plots）

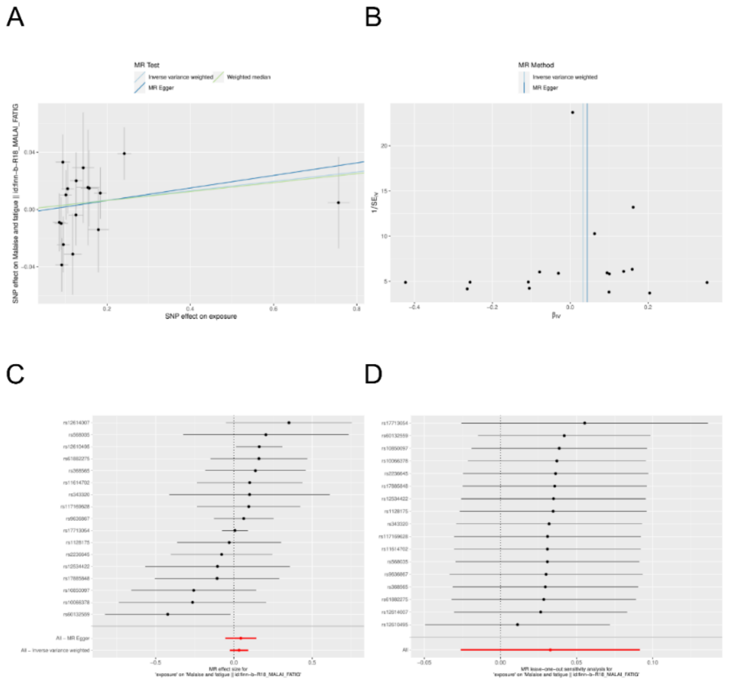

Figure.S1.3 Results of univariate Mendelian randomization of Very severe respiratory confirmed covid-Malaise and fatigue.

(A.scatter plots of causality, B. funnel plots, C.forest plots of each SNPs, D.leave-one-out plots）

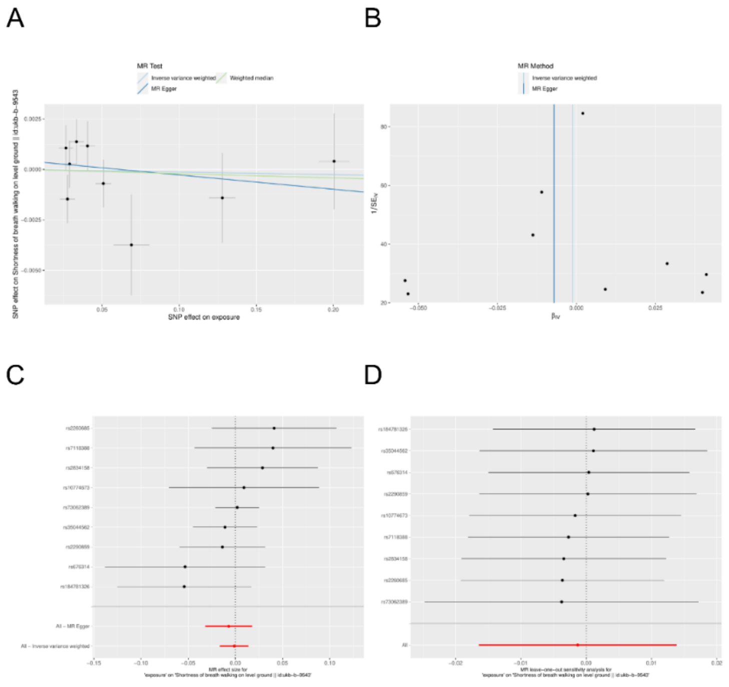

Figure.S2.1 Results of univariate Mendelian randomization of covid-19- Shortness of breath walking on level ground.

(A.scatter plots of causality, B. funnel plots, C.forest plots of each SNPs, D.leave-one-out plots）

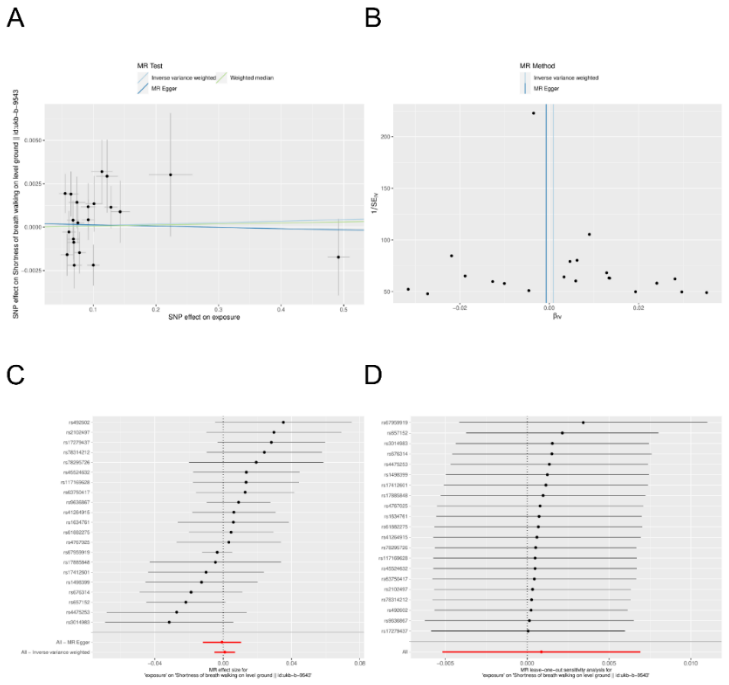

Figure.S2.2 Results of univariate Mendelian randomization of Hospitalized covid-Shortness of breath walking on level ground.

(A.scatter plots of causality, B. funnel plots, C.forest plots of each SNPs, D.leave-one-out plots）

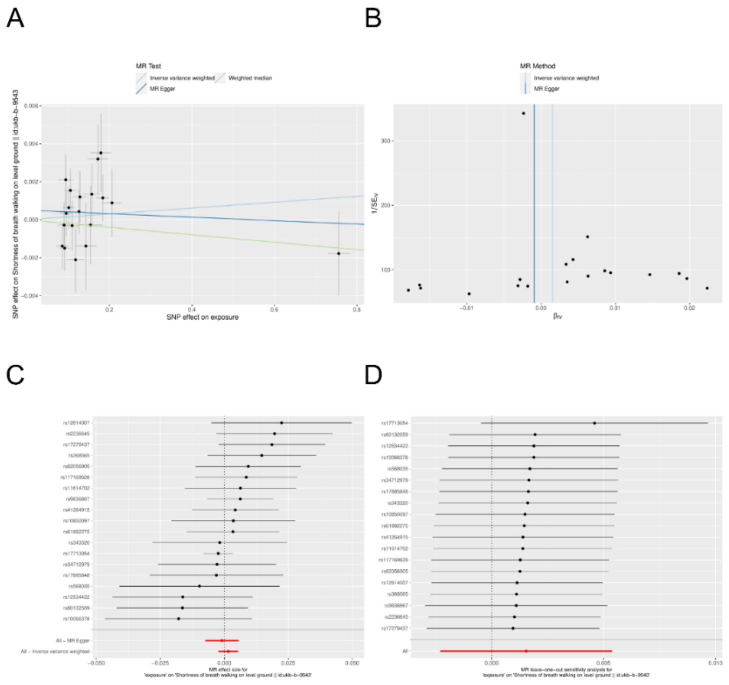

Figure.S2.3 Results of univariate Mendelian randomization of Very severe respiratory confirmed covid-Shortness of breath walking on level ground.

(A.scatter plots of causality, B. funnel plots, C.forest plots of each SNPs, D.leave-one-out plots）

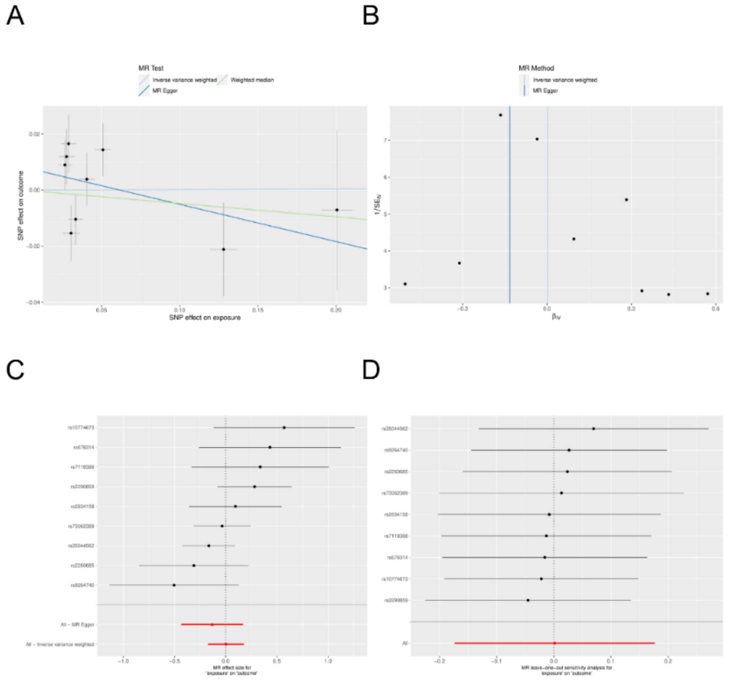

Figure.S3.1 Results of univariate Mendelian randomization of covid-19- Heart arrhythmia.
(A.scatter plots of causality, B. funnel plots, C.forest plots of each SNPs, D.leave-one-out plots）

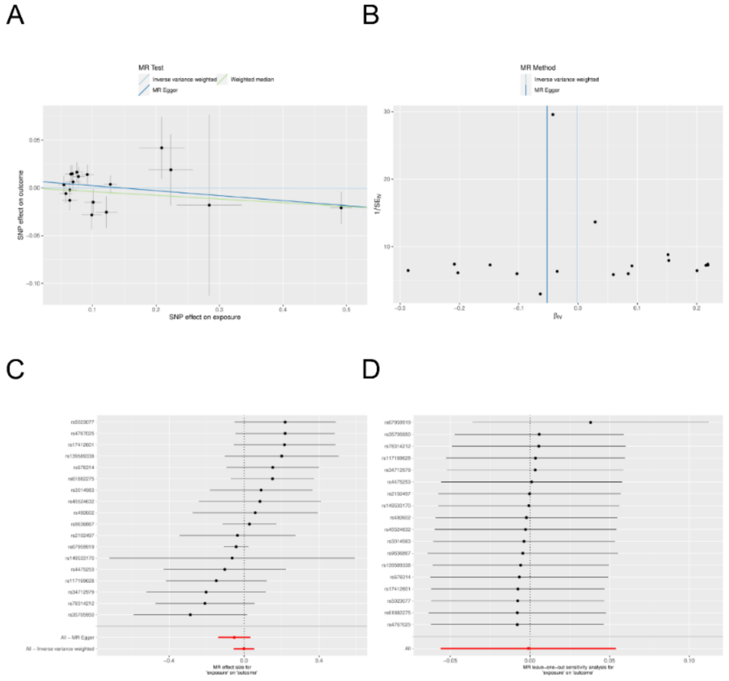

Figure.S3.2 Results of univariate Mendelian randomization of Hospitalized covid-Shortness of breath walking on level ground.
(A.scatter plots of causality, B. funnel plots, C.forest plots of each SNPs, D.leave-one-out plots）

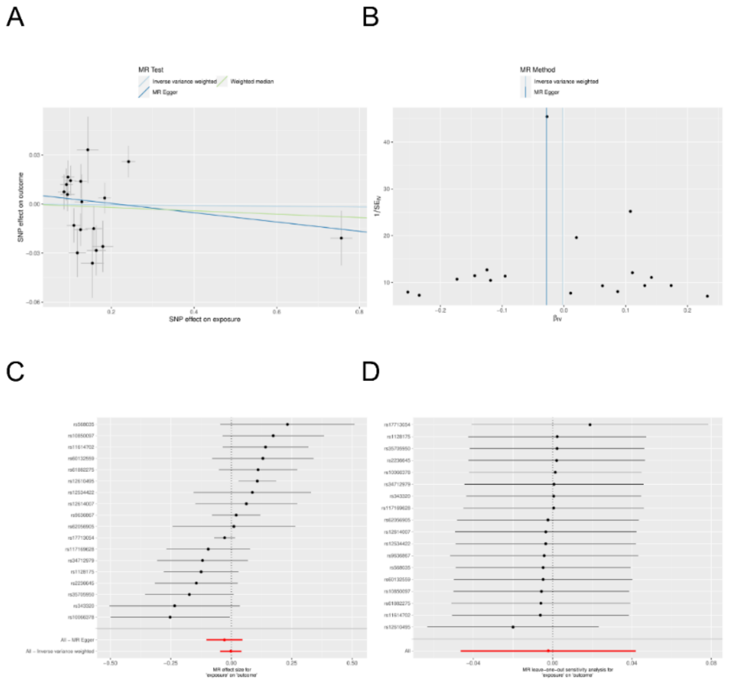

Figure.S3.3 Results of univariate Mendelian randomization of Very severe respiratory confirmed covid-Shortness of breath walking on level ground.

(A.scatter plots of causality, B. funnel plots, C.forest plots of each SNPs, D.leave-one-out plots）

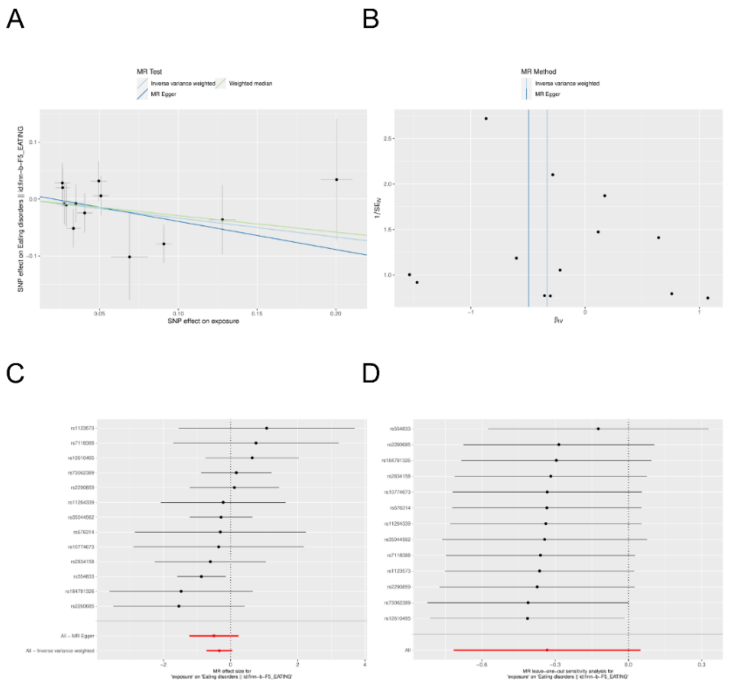

Figure.S4.1 Results of univariate Mendelian randomization of covid-19- Eating disorders.

(A.scatter plots of causality, B. funnel plots, C.forest plots of each SNPs, D.leave-one-out plots）

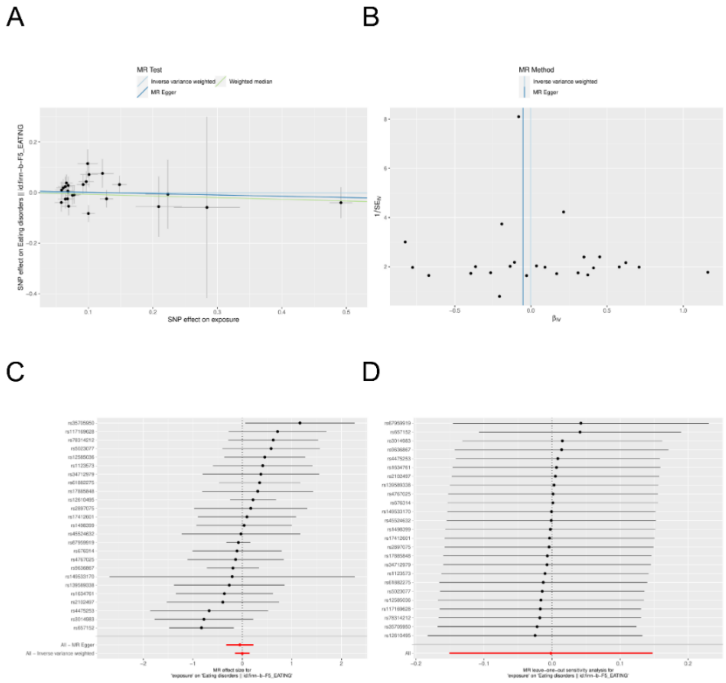

Figure.S4.2 Results of univariate Mendelian randomization of Hospitalized covid- Eating disorders.
(A.scatter plots of causality, B. funnel plots, C.forest plots of each SNPs, D.leave-one-out plots）

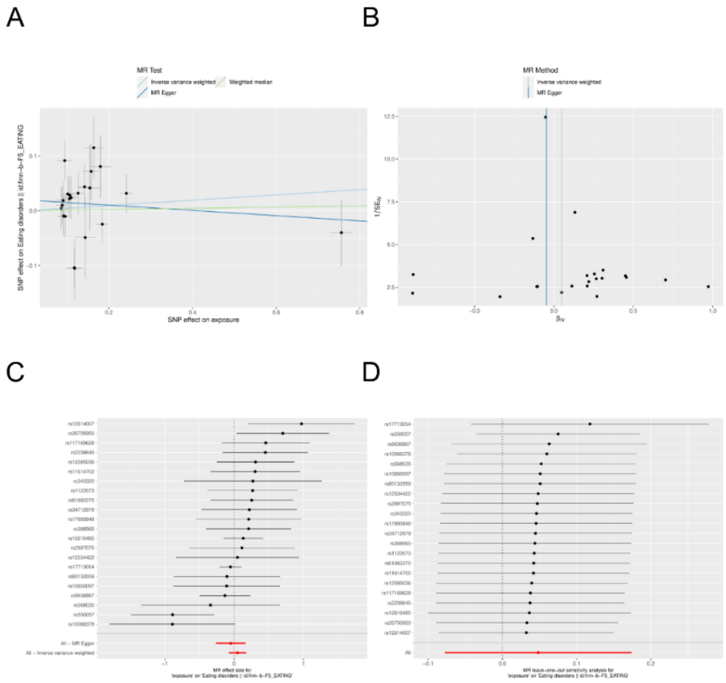

Figure.S4.3 Results of univariate Mendelian randomization of Very severe respiratory confirmed covid-Shortness of breath walking on level ground.
(A.scatter plots of causality, B. funnel plots, C.forest plots of each SNPs, D.leave-one-out plots）

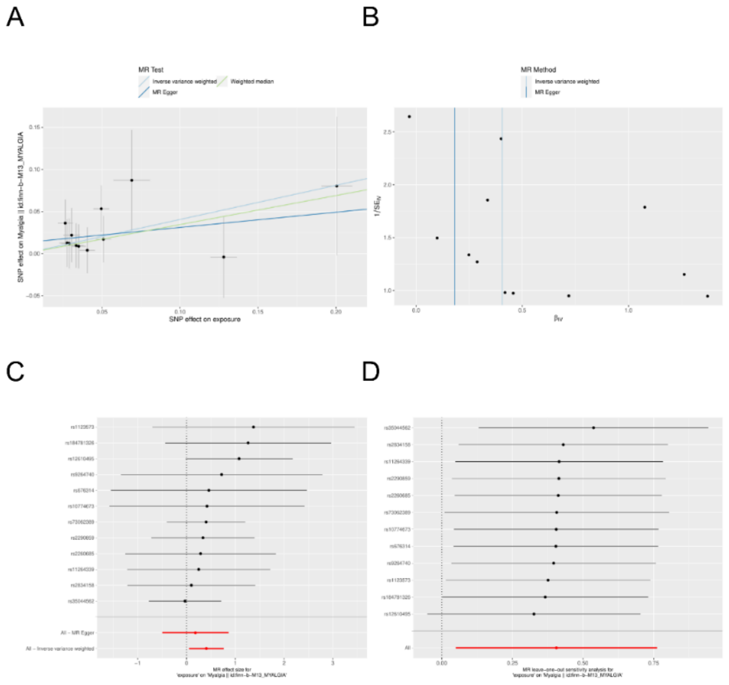

Figure.S5.1 Results of univariate Mendelian randomization of covid-19-Myalgia.
(A.scatter plots of causality, B. funnel plots, C.forest plots of each SNPs, D.leave-one-out plots）

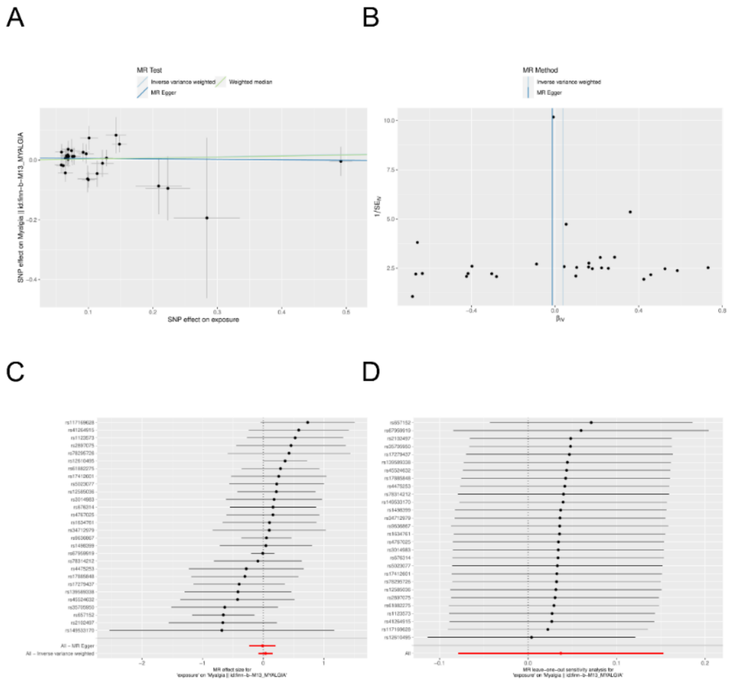

Figure.S5.2 Results of univariate Mendelian randomization of Hospitalized covid-Myalgia.
(A.scatter plots of causality, B. funnel plots, C.forest plots of each SNPs, D.leave-one-out plots）

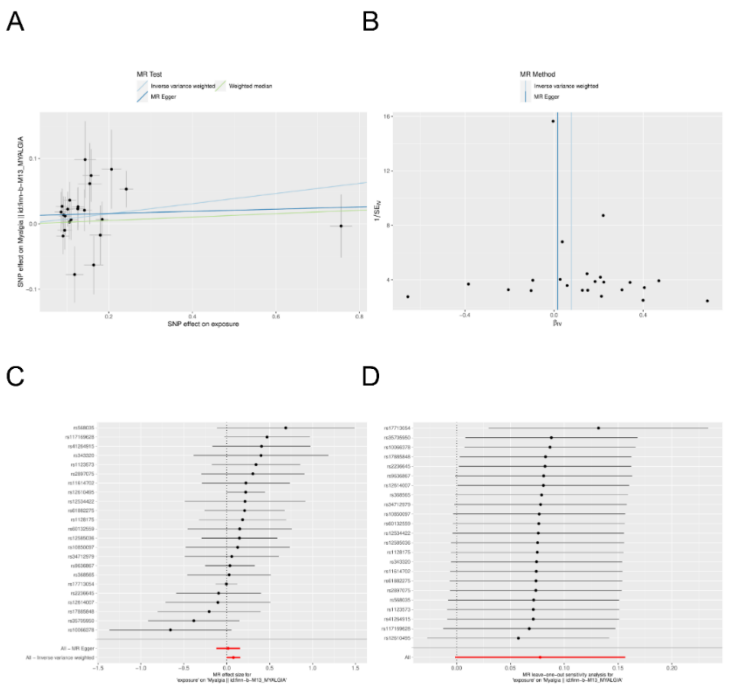

Figure.S5.3 Results of univariate Mendelian randomization of Very severe respiratory confirmed covid- Myalgia.

(A.scatter plots of causality, B. funnel plots, C.forest plots of each SNPs, D.leave-one-out plots）

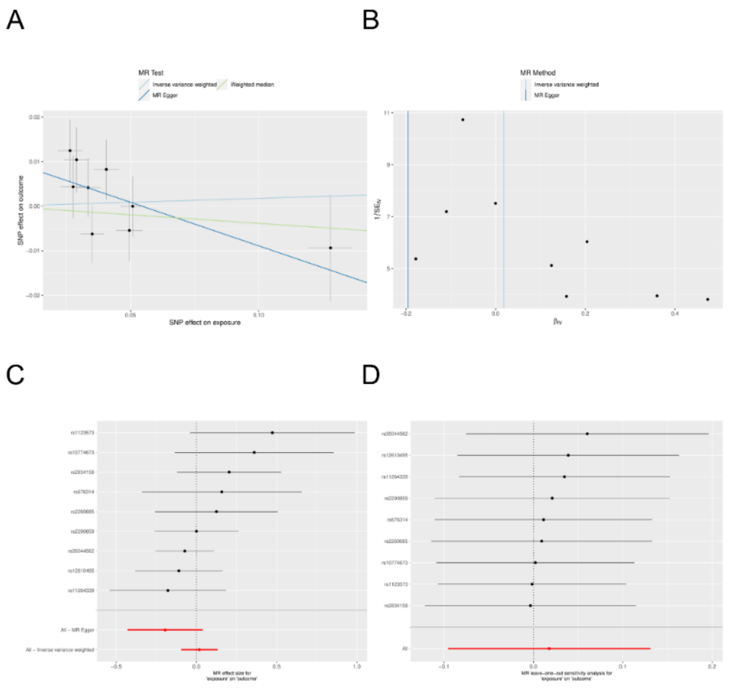

Figure.S6.1 Results of univariate Mendelian randomization of covid-19- Dorsalgia.

(A.scatter plots of causality, B. funnel plots, C.forest plots of each SNPs, D.leave-one-out plots）

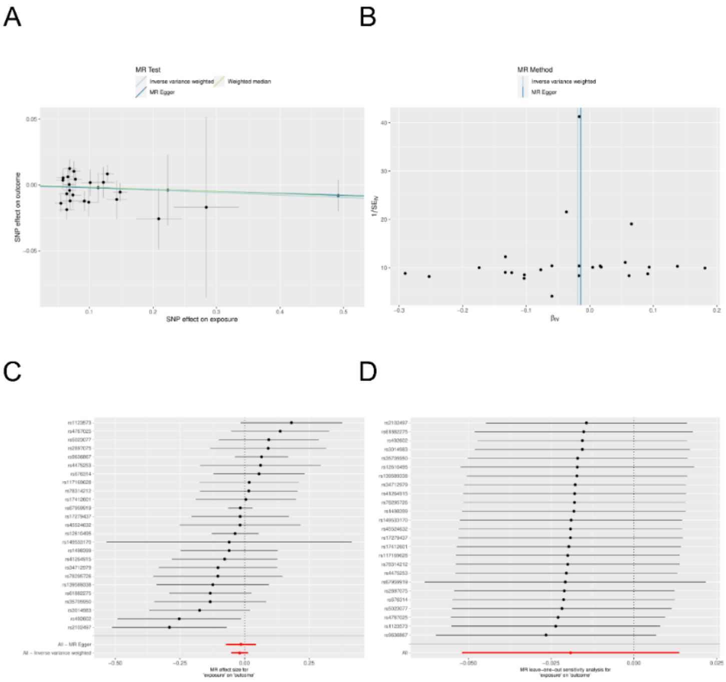

Figure.S6.2 Results of univariate Mendelian randomization of Hospitalized covid- Dorsalgia.
(A.scatter plots of causality, B. funnel plots, C.forest plots of each SNPs, D.leave-one-out plots）

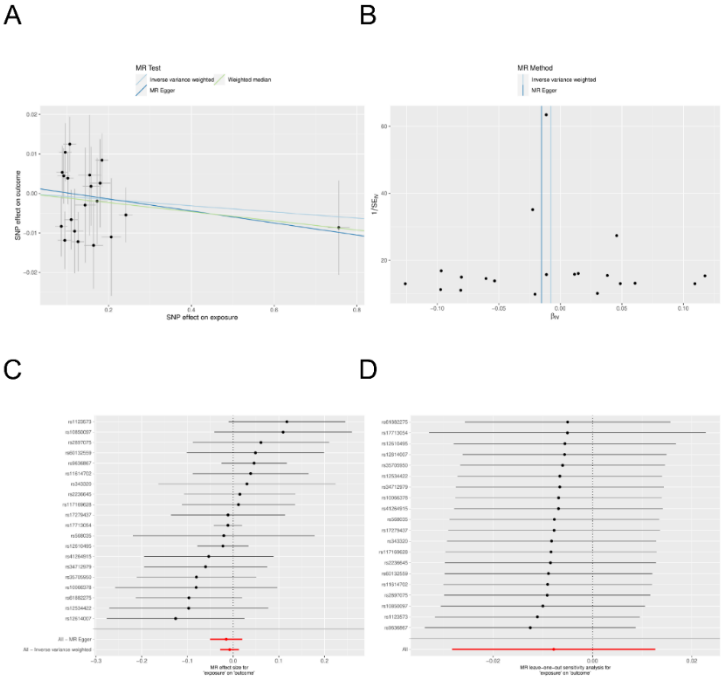

Figure.S6.3 Results of univariate Mendelian randomization of Very severe respiratory confirmed covid- Dorsalgia.
(A.scatter plots of causality, B. funnel plots, C.forest plots of each SNPs, D.leave-one-out plots）

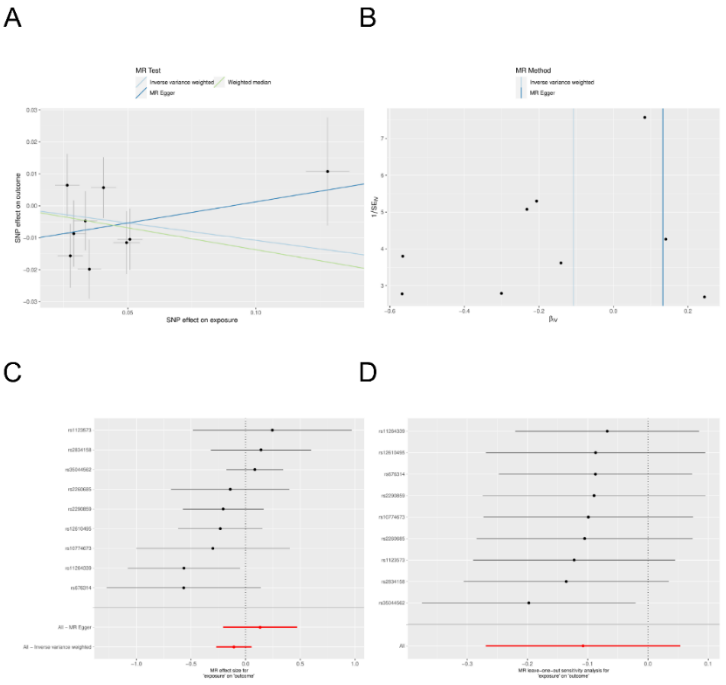

Figure.S7.1 Results of univariate Mendelian randomization of covid-19- Joint pain.
(A.scatter plots of causality, B. funnel plots, C.forest plots of each SNPs, D.leave-one-out plots）

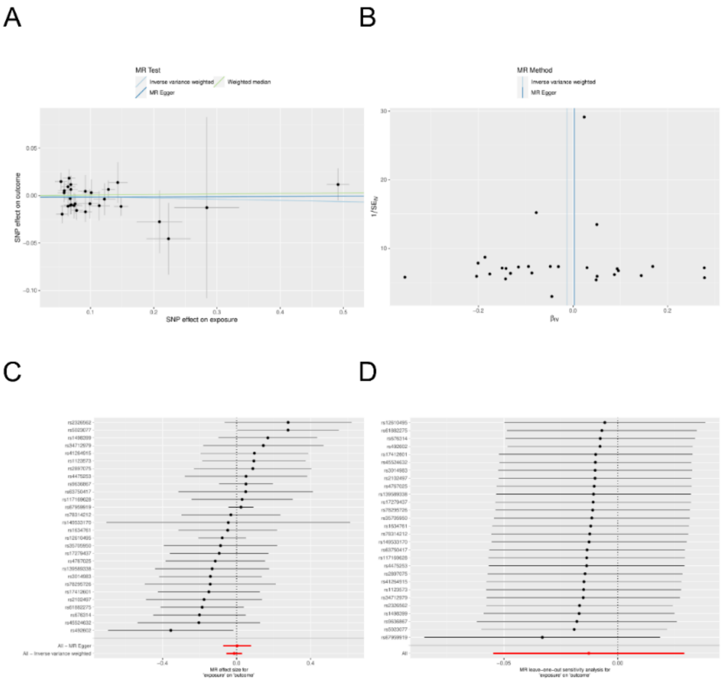

Figure.S7.2 Results of univariate Mendelian randomization of Hospitalized covid- Joint pain.
(A.scatter plots of causality, B. funnel plots, C.forest plots of each SNPs, D.leave-one-out plots）

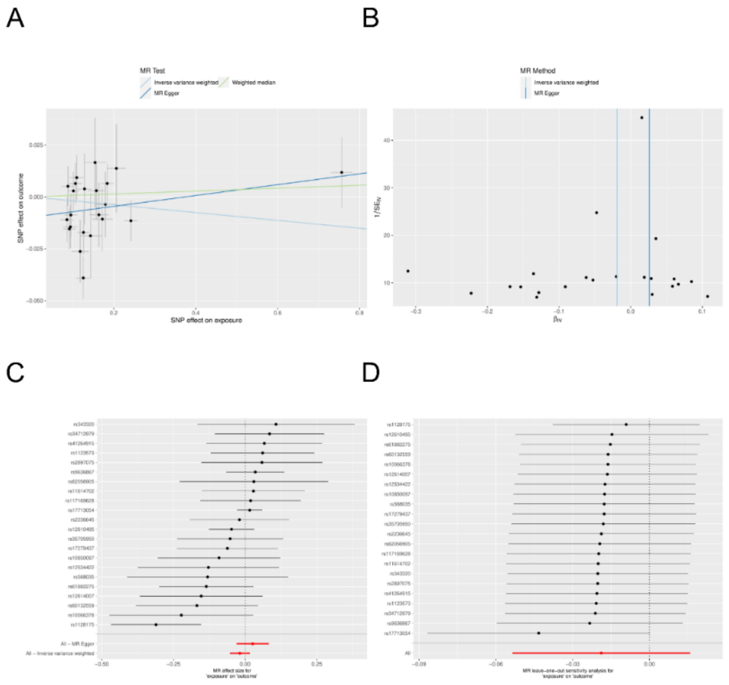

Figure.S7.3 Results of univariate Mendelian randomization of Very severe respiratory confirmed covid- Joint pain.

(A.scatter plots of causality, B. funnel plots, C.forest plots of each SNPs, D.leave-one-out plots）

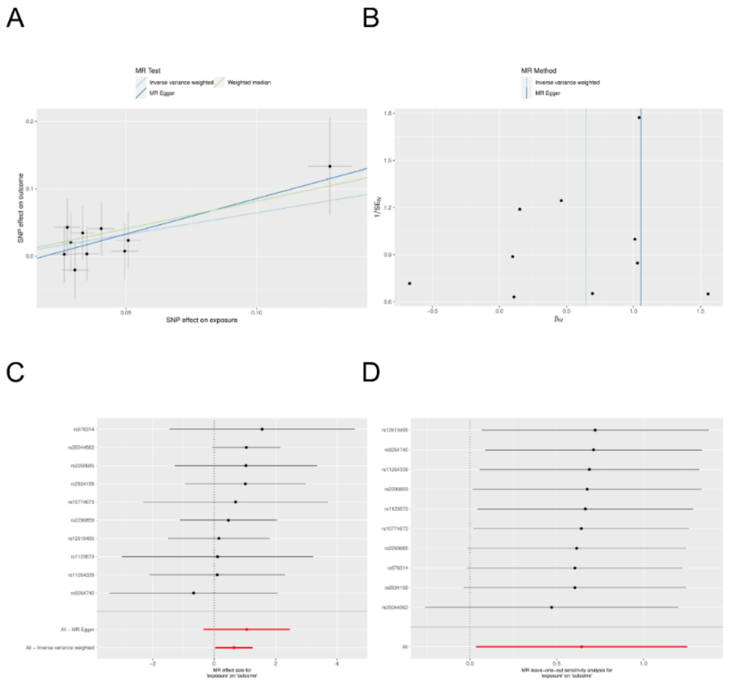

Figure.S8.1 Results of univariate Mendelian randomization of covid-19- Pain (limb, back, neck, head abdominally).

(A.scatter plots of causality, B. funnel plots, C.forest plots of each SNPs, D.leave-one-out plots）

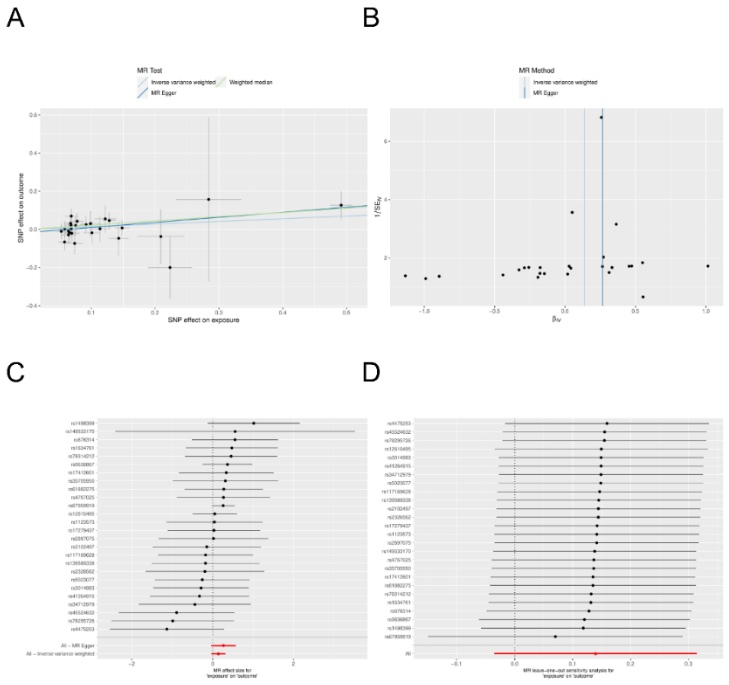

Figure.S8.2 Results of univariate Mendelian randomization of Hospitalized covid- Pain (limb, back, neck, head abdominally).

(A.scatter plots of causality, B. funnel plots, C.forest plots of each SNPs, D.leave-one-out plots）

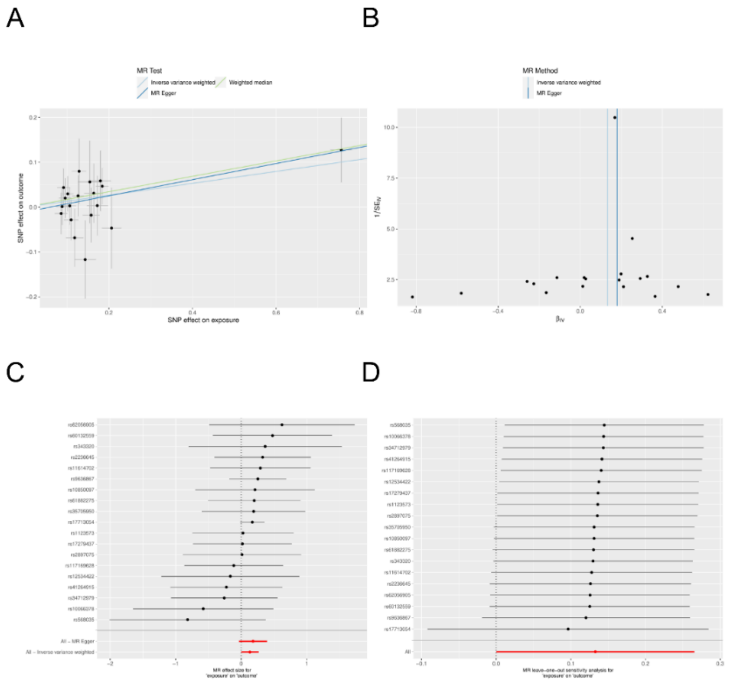

Figure.S8.3 Results of univariate Mendelian randomization of Very severe respiratory confirmed covid- Pain (limb, back, neck, head abdominally).

(A.scatter plots of causality, B. funnel plots, C.forest plots of each SNPs, D.leave-one-out plots）

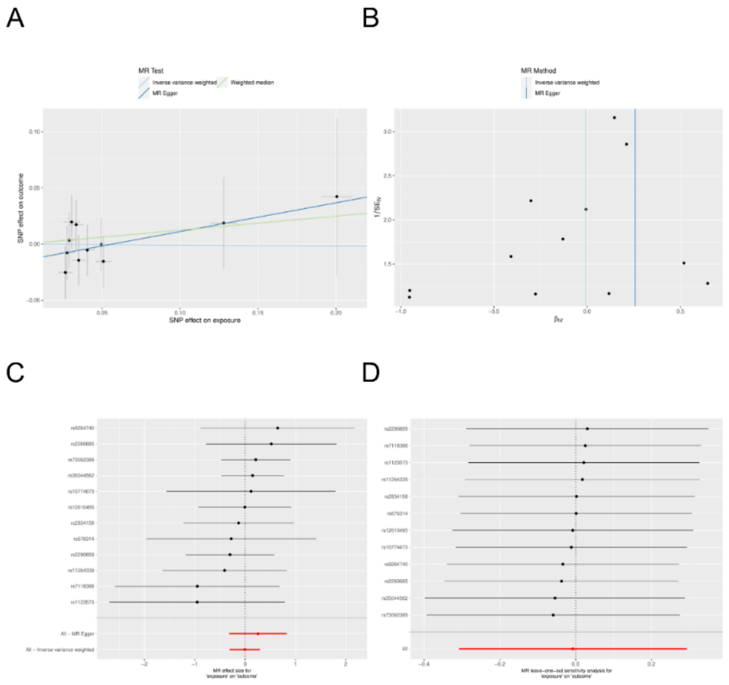

Figure.S9.1 Results of univariate Mendelian randomization of covid-19- Sleeplessness / insomnia.
(A.scatter plots of causality, B. funnel plots, C.forest plots of each SNPs, D.leave-one-out plots）

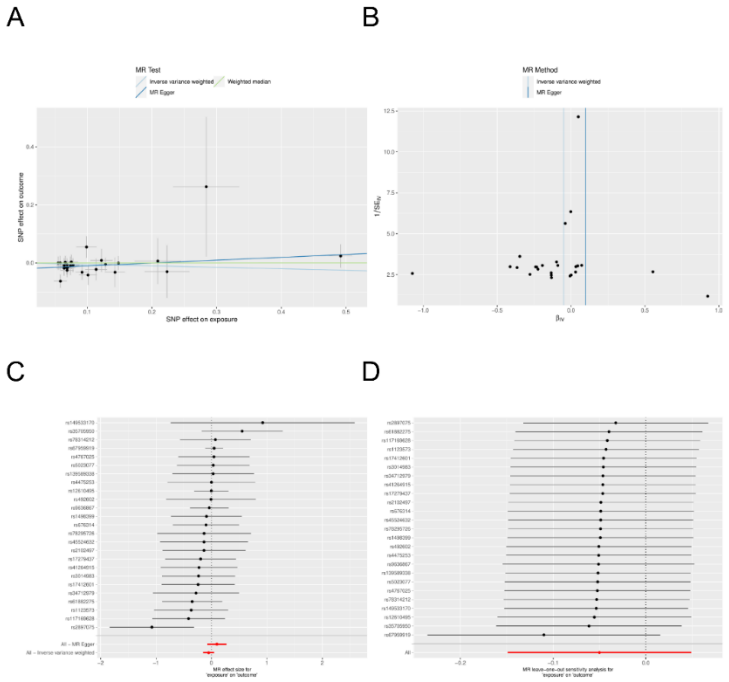

Figure.S9.2 Results of univariate Mendelian randomization of Hospitalized covid- Sleeplessness / insomnia.
(A.scatter plots of causality, B. funnel plots, C.forest plots of each SNPs, D.leave-one-out plots）

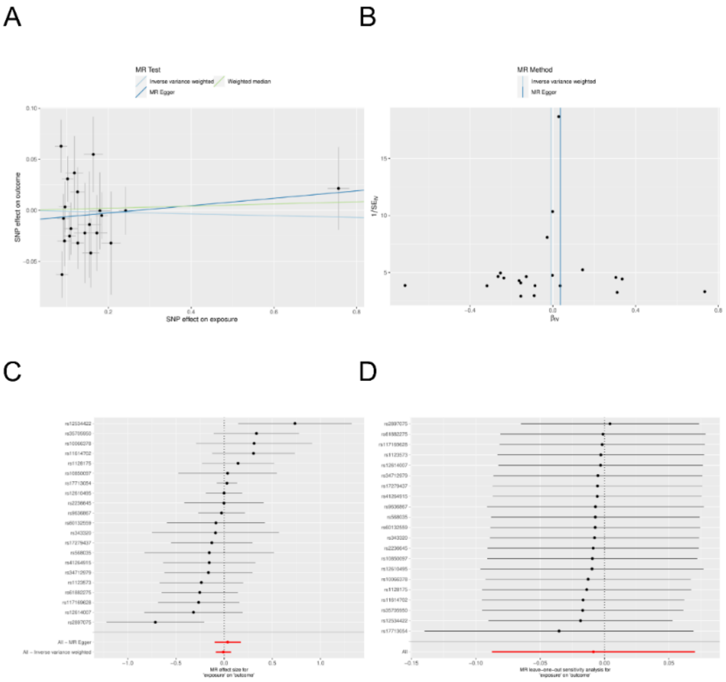

Figure.S9.3 Results of univariate Mendelian randomization of Very severe respiratory confirmed covid- Sleeplessness / insomnia.

(A.scatter plots of causality, B. funnel plots, C.forest plots of each SNPs, D.leave-one-out plots）

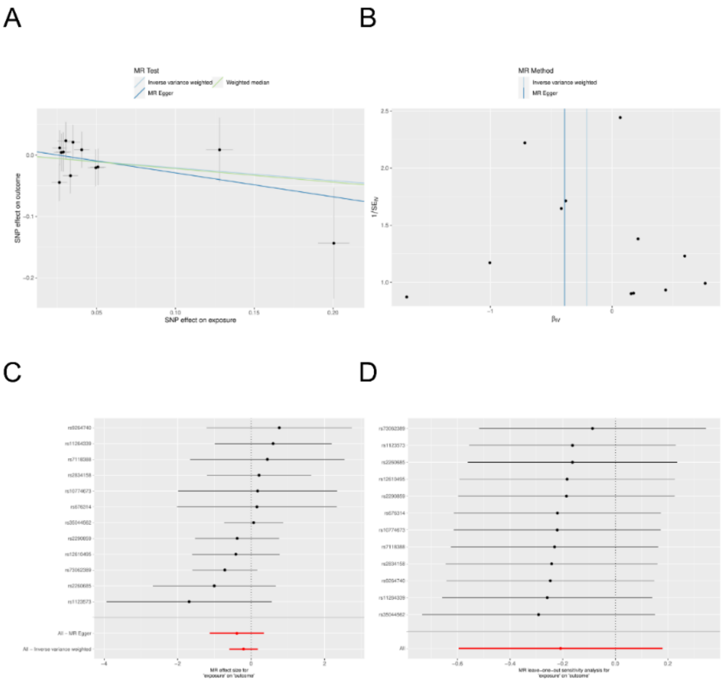

Figure.S10.1 Results of univariate Mendelian randomization of covid-19- Memory loss.

(A.scatter plots of causality, B. funnel plots, C.forest plots of each SNPs, D.leave-one-out plots）

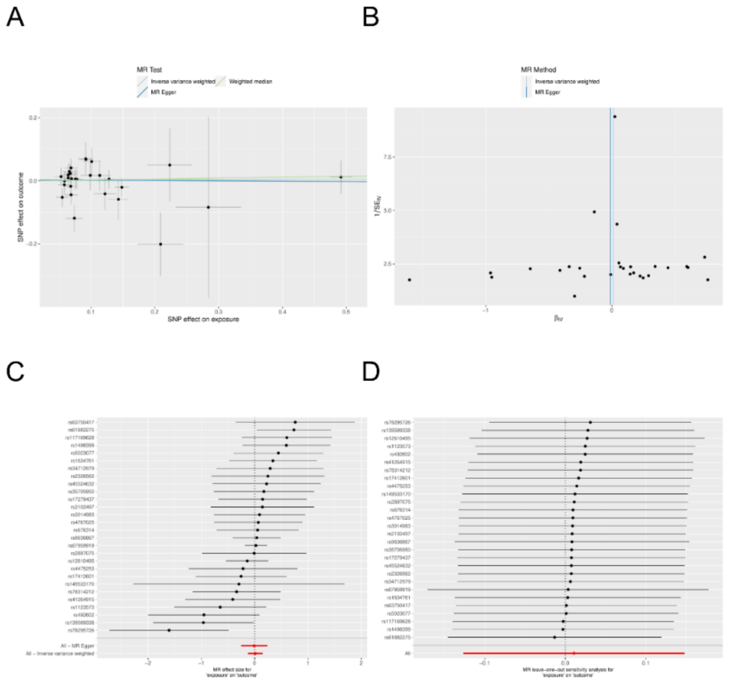

Figure.S10.2 Results of univariate Mendelian randomization of Hospitalized covid- Memory loss.
(A.scatter plots of causality, B. funnel plots, C.forest plots of each SNPs, D.leave-one-out plots）

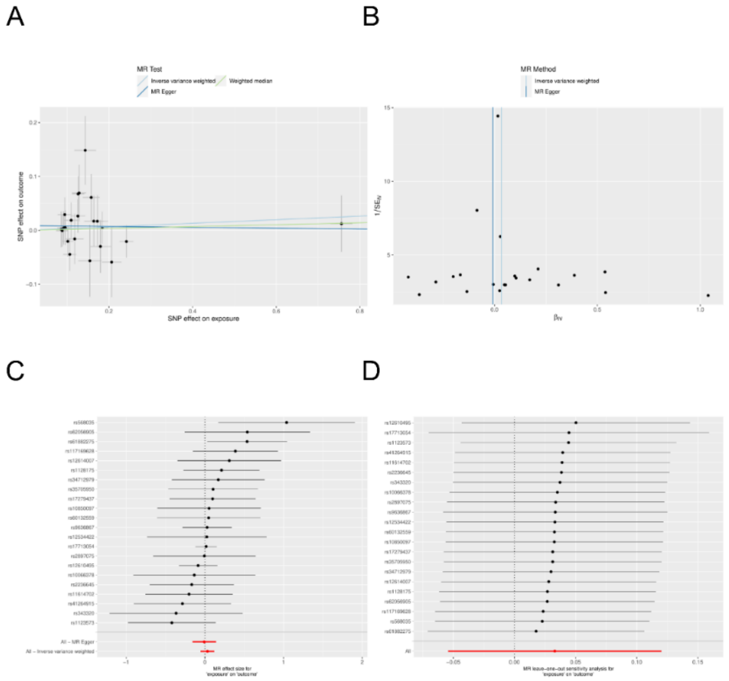

Figure.S10.3 Results of univariate Mendelian randomization of Very severe respiratory confirmed covid - Memory loss.
(A.scatter plots of causality, B. funnel plots, C.forest plots of each SNPs, D.leave-one-out plots）

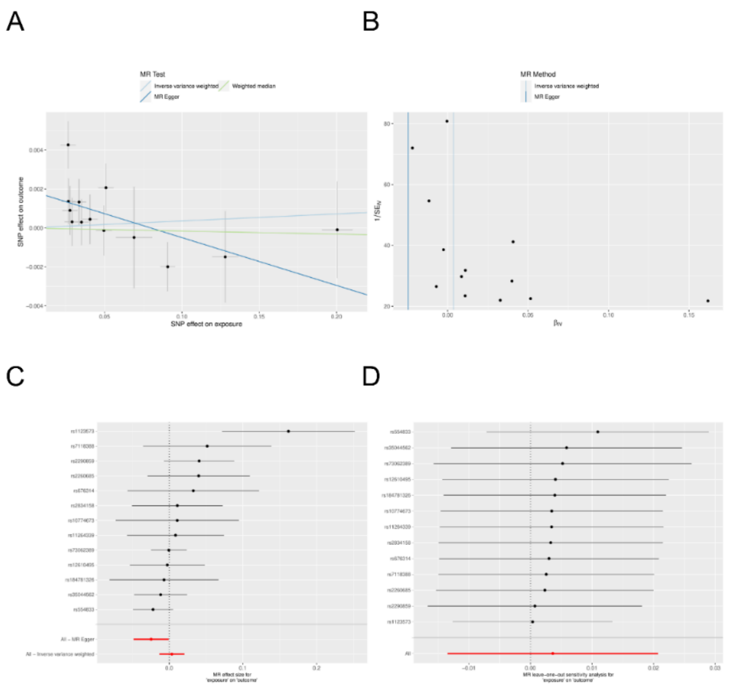

Figure.S11.1 Results of univariate Mendelian randomization of covid-19- Depression (broad).
(A.scatter plots of causality, B. funnel plots, C.forest plots of each SNPs, D.leave-one-out plots）

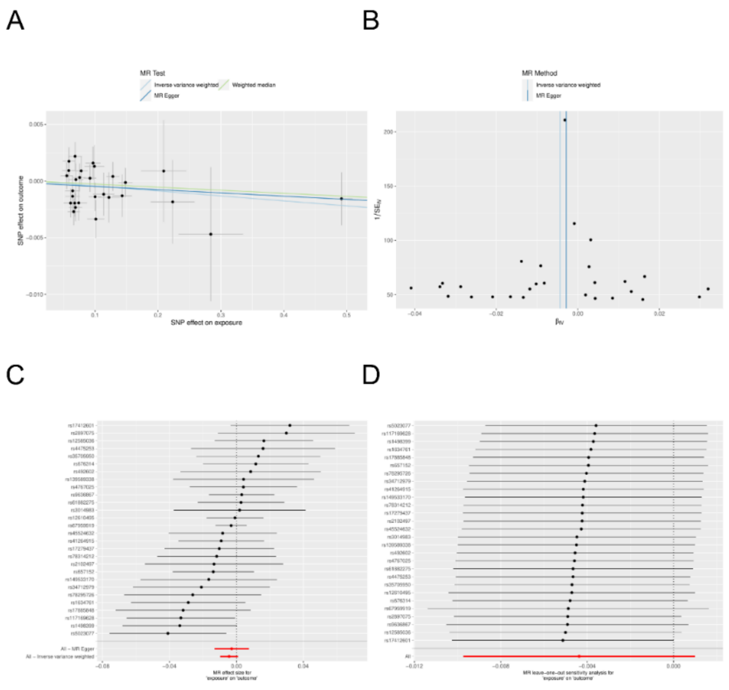

Figure.S11.2 Results of univariate Mendelian randomization of Hospitalized covid - Depression (broad).
(A.scatter plots of causality, B. funnel plots, C.forest plots of each SNPs, D.leave-one-out plots）

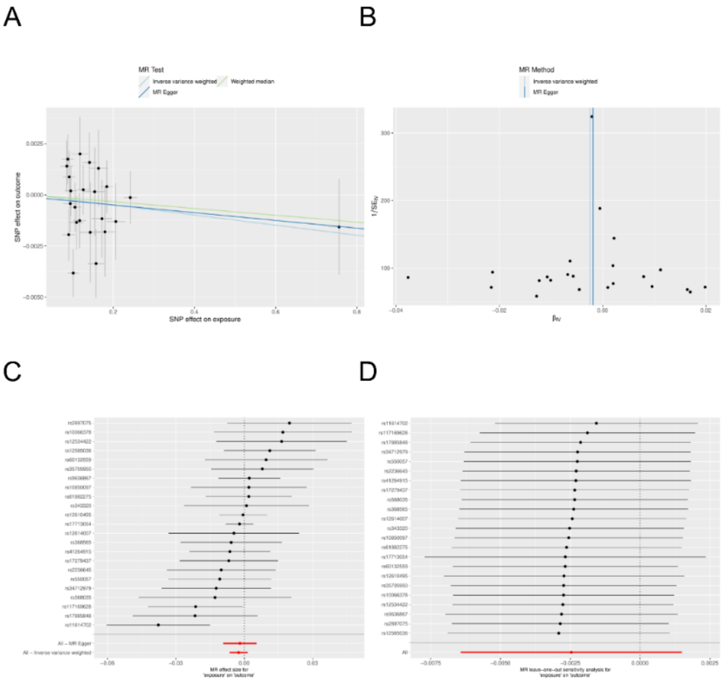

Figure.S11.3 Results of univariate Mendelian randomization of Very severe respiratory confirmed covid-Depression (broad).

(A.scatter plots of causality, B. funnel plots, C.forest plots of each SNPs, D.leave-one-out plots）

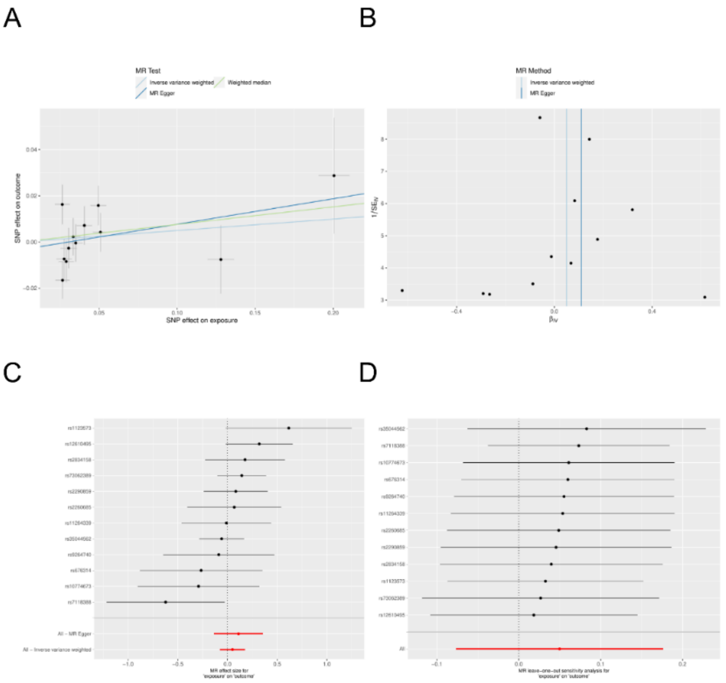

Figure.S12.1 Results of univariate Mendelian randomization of covid-19- Anxiety.

(A.scatter plots of causality, B. funnel plots, C.forest plots of each SNPs, D.leave-one-out plots）

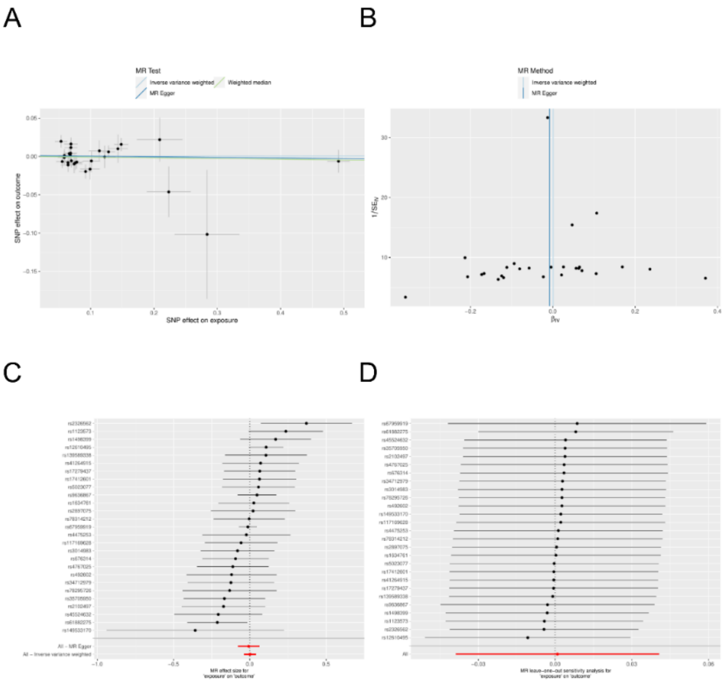

Figure.S12.2 Results of univariate Mendelian randomization of Hospitalized covid - Anxiety.
(A.scatter plots of causality, B. funnel plots, C.forest plots of each SNPs, D.leave-one-out plots）

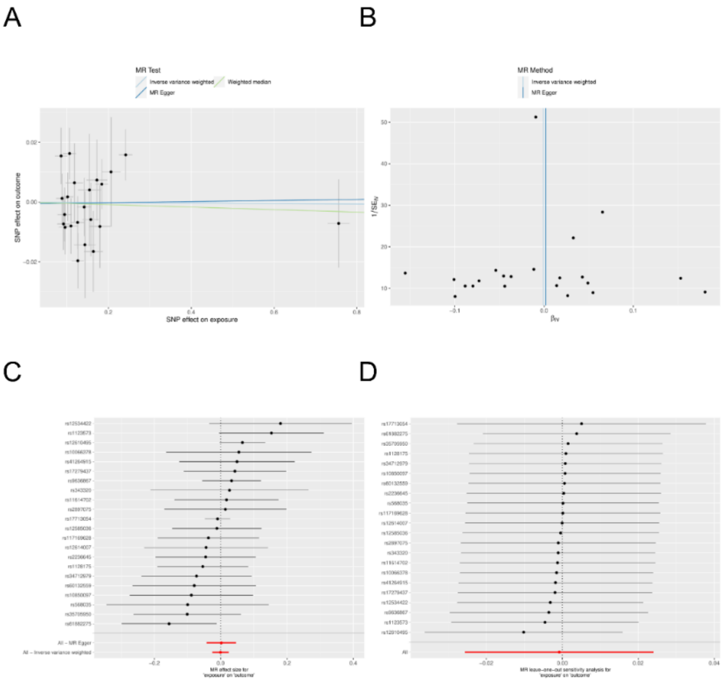

Figure.S12.3 Results of univariate Mendelian randomization of Very severe respiratory confirmed covid- Anxiety.
(A.scatter plots of causality, B. funnel plots, C.forest plots of each SNPs, D.leave-one-out plots）

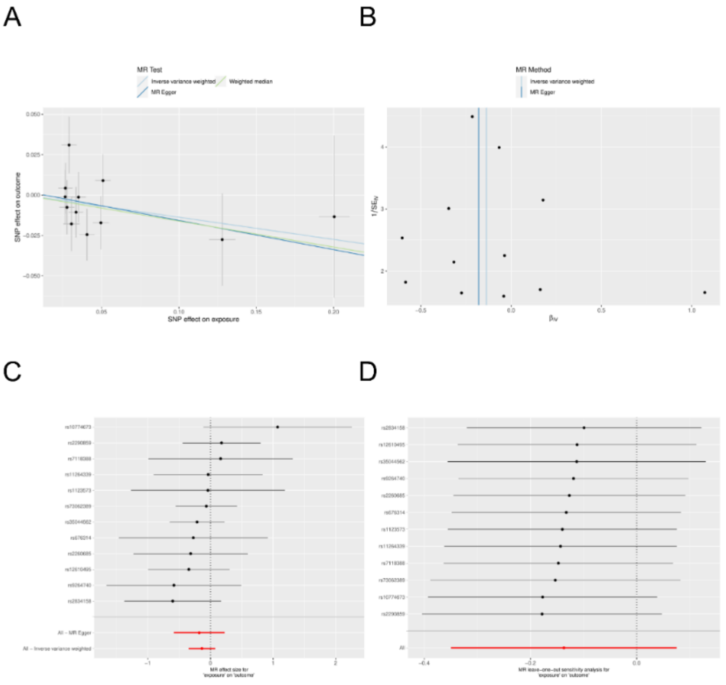

Figure.S13.1 Results of univariate Mendelian randomization of covid-19- Ever attempted suicide.

(A.scatter plots of causality, B. funnel plots, C.forest plots of each SNPs, D.leave-one-out plots）

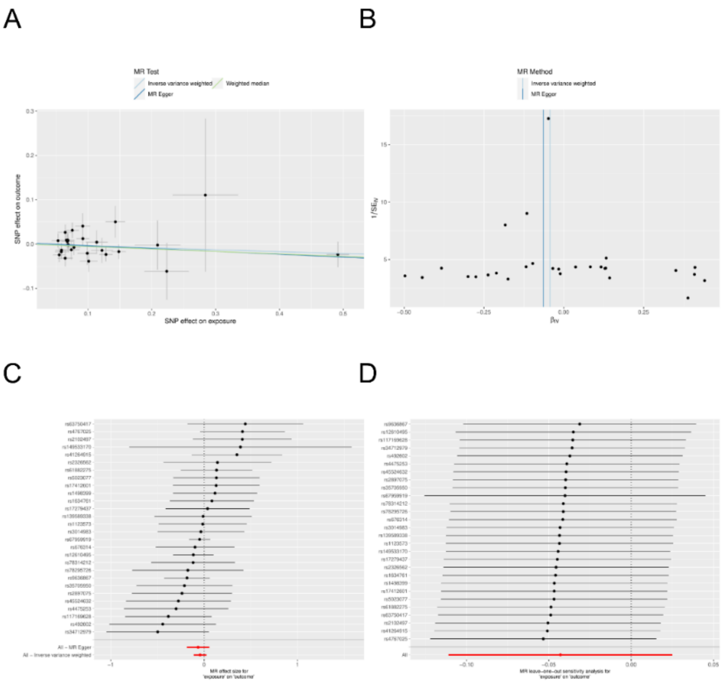

Figure.S13.2 Results of univariate Mendelian randomization of Hospitalized covid - Ever attempted suicide.

(A.scatter plots of causality, B. funnel plots, C.forest plots of each SNPs, D.leave-one-out plots）

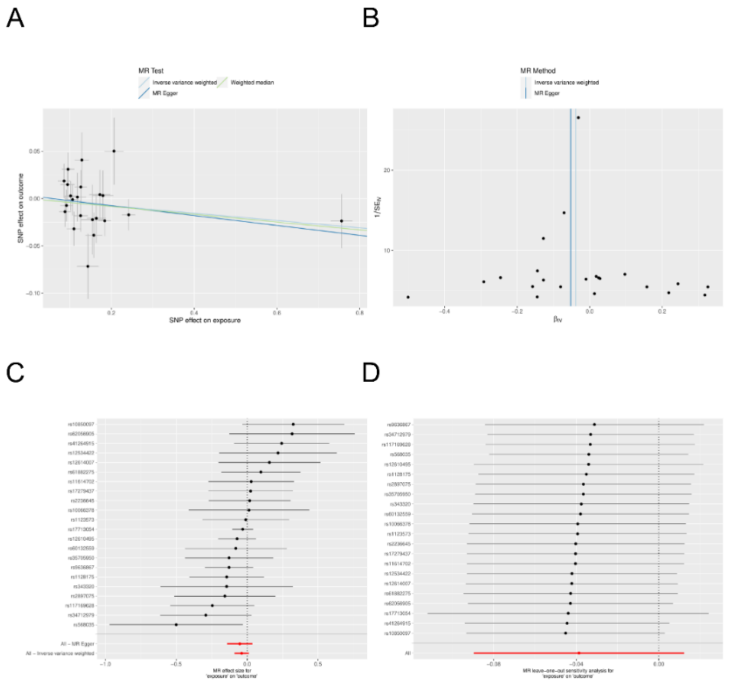

Figure.S13.3 Results of univariate Mendelian randomization of Very severe respiratory confirmed covid- Ever attempted suicide.
(A.scatter plots of causality, B. funnel plots, C.forest plots of each SNPs, D.leave-one-out plots）

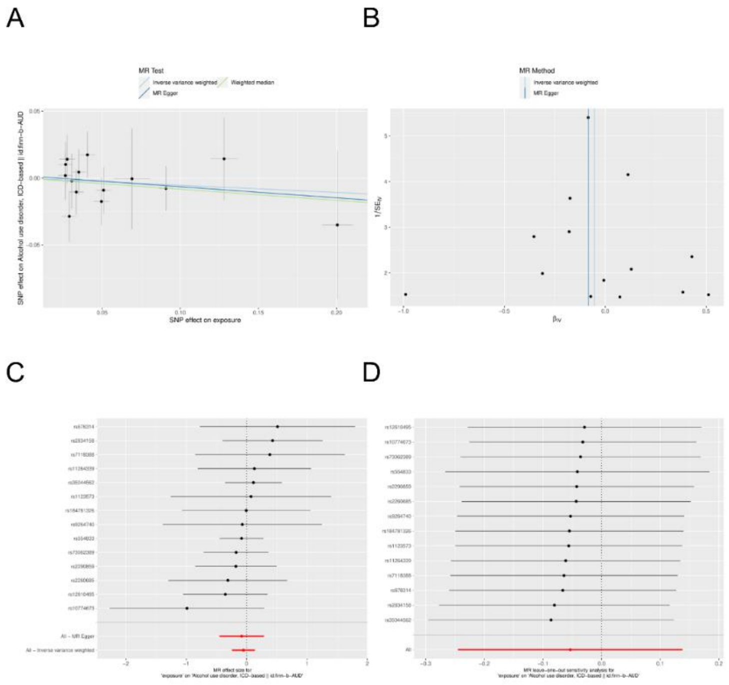

Figure.S14.1 Results of univariate Mendelian randomization of covid-19- Alcohol use.
(A.scatter plots of causality, B. funnel plots, C.forest plots of each SNPs, D.leave-one-out plots）

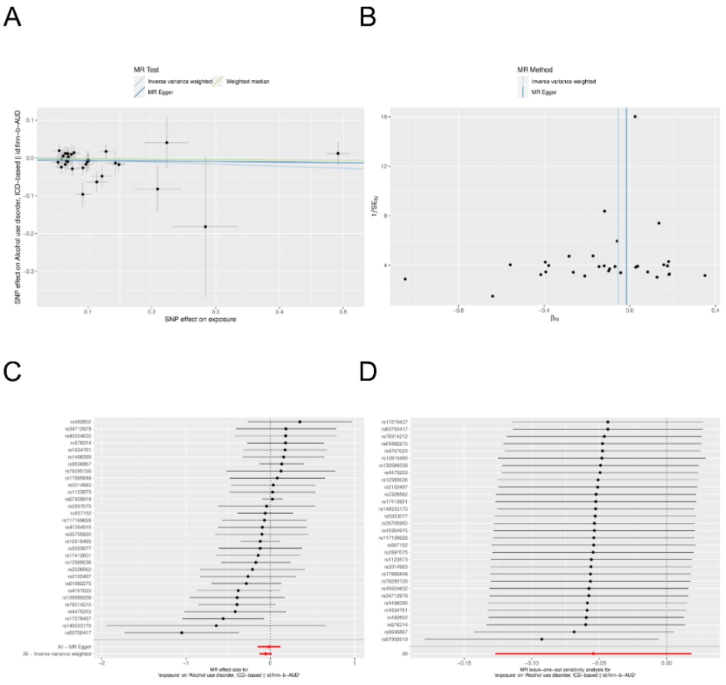

Figure.S14.2 Results of univariate Mendelian randomization of Hospitalized covid - Alcohol use.

(A.scatter plots of causality, B. funnel plots, C.forest plots of each SNPs, D.leave-one-out plots）

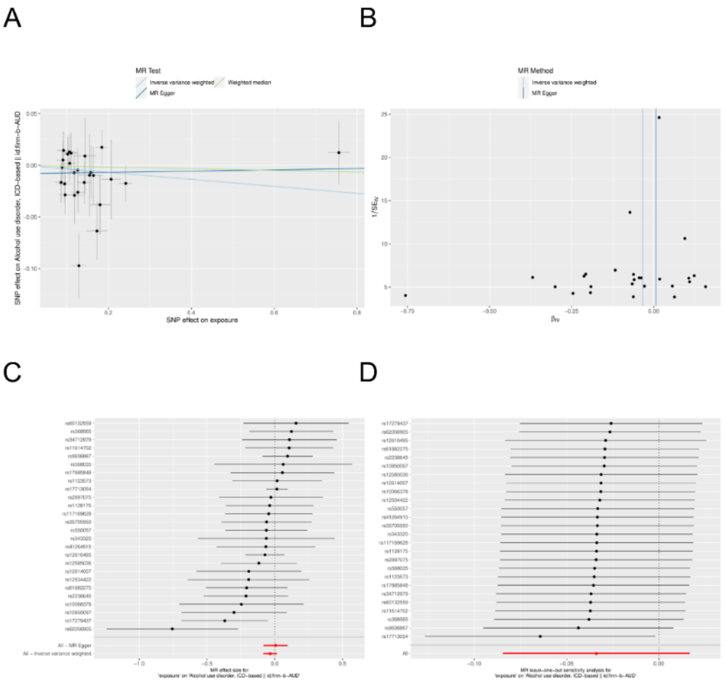

Figure.S14.3 Results of univariate Mendelian randomization of Very severe respiratory confirmed covid- Alcohol use.

(A.scatter plots of causality, B. funnel plots, C.forest plots of each SNPs, D.leave-one-out plots）

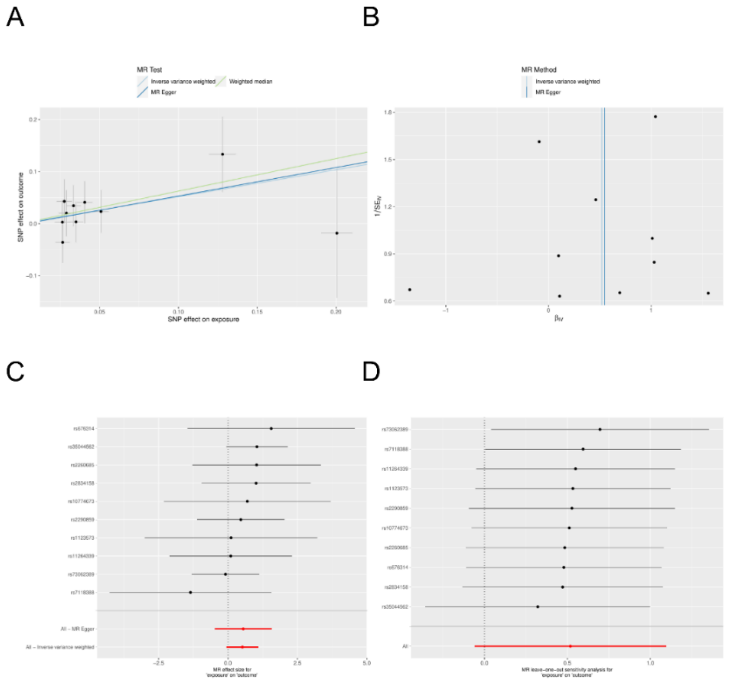

Figure.S15.1 Results of univariate Mendelian randomization of covid-19- Tobacco use.
(A.scatter plots of causality, B. funnel plots, C.forest plots of each SNPs, D.leave-one-out plots)

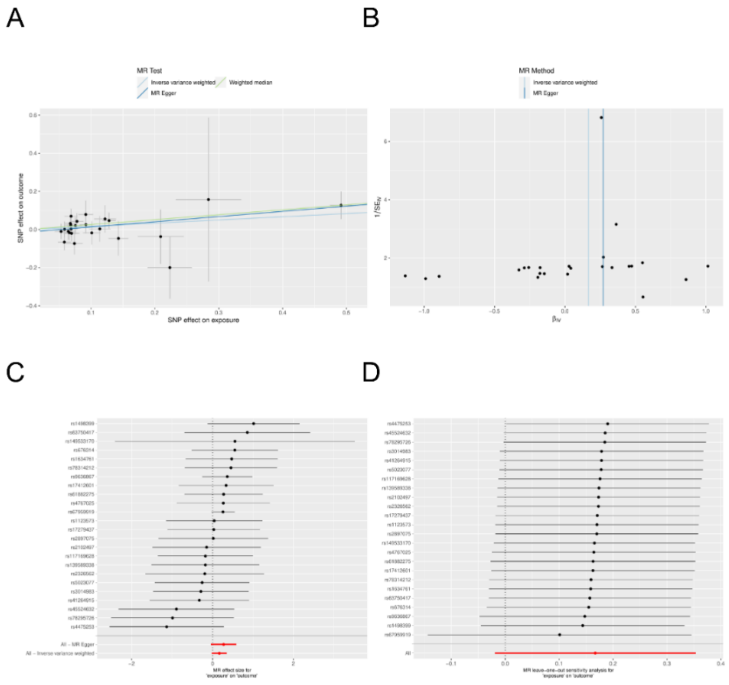

Figure.S15.2 Results of univariate Mendelian randomization of Hospitalized covid - Tobacco use.
(A.scatter plots of causality, B. funnel plots, C.forest plots of each SNPs, D.leave-one-out plots)

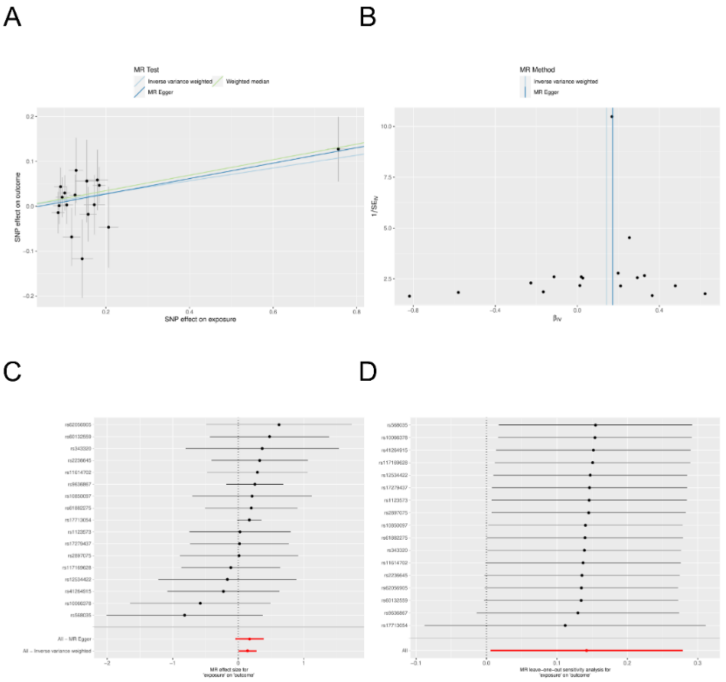

Figure.S15.3 Results of univariate Mendelian randomization of Very severe respiratory confirmed covid- Tobacco use.
(A.scatter plots of causality, B. funnel plots, C.forest plots of each SNPs, D.leave-one-out plots）

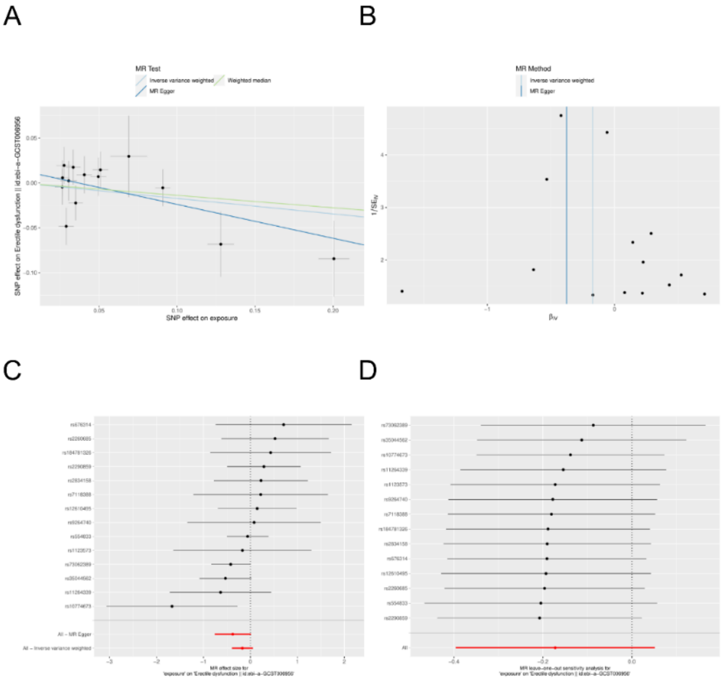

Figure.S16.1 Results of univariate Mendelian randomization of covid-19- Erectile dysfunction.
(A.scatter plots of causality, B. funnel plots, C.forest plots of each SNPs, D.leave-one-out plots）

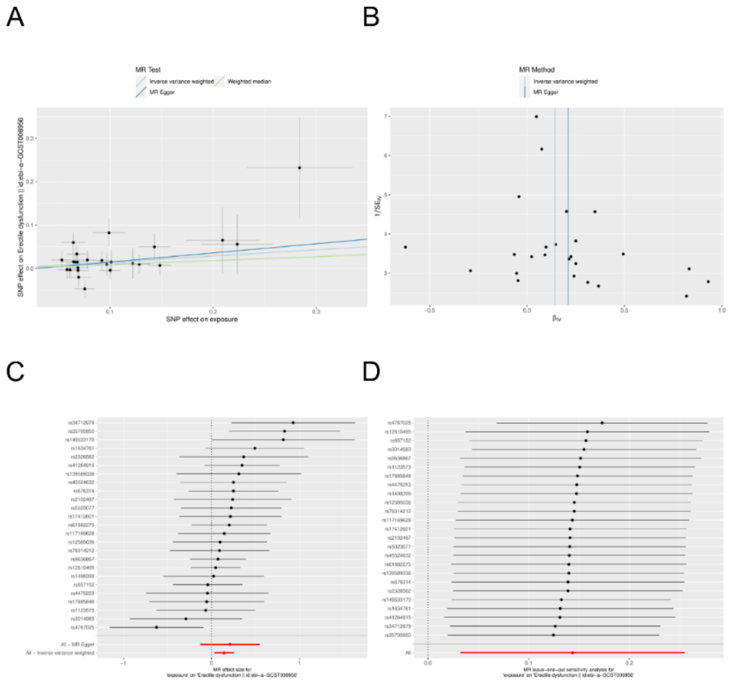

Figure.S16.2 Results of univariate Mendelian randomization of Hospitalized covid- Erectile dysfunction.

(A.scatter plots of causality, B. funnel plots, C.forest plots of each SNPs, D.leave-one-out plots）

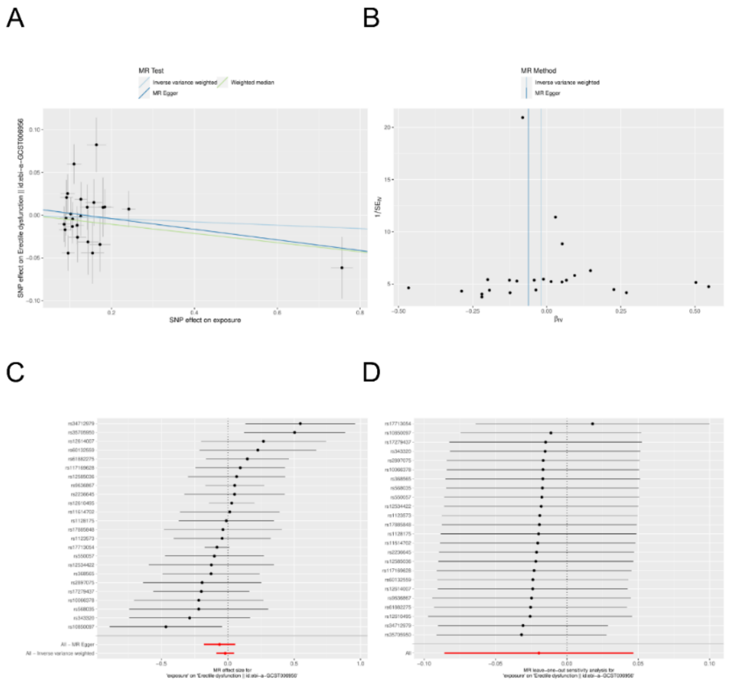

Figure.S16.3 Results of univariate Mendelian randomization of Very severe respiratory confirmed covid- Erectile dysfunction.

(A.scatter plots of causality, B. funnel plots, C.forest plots of each SNPs, D.leave-one-out plots）

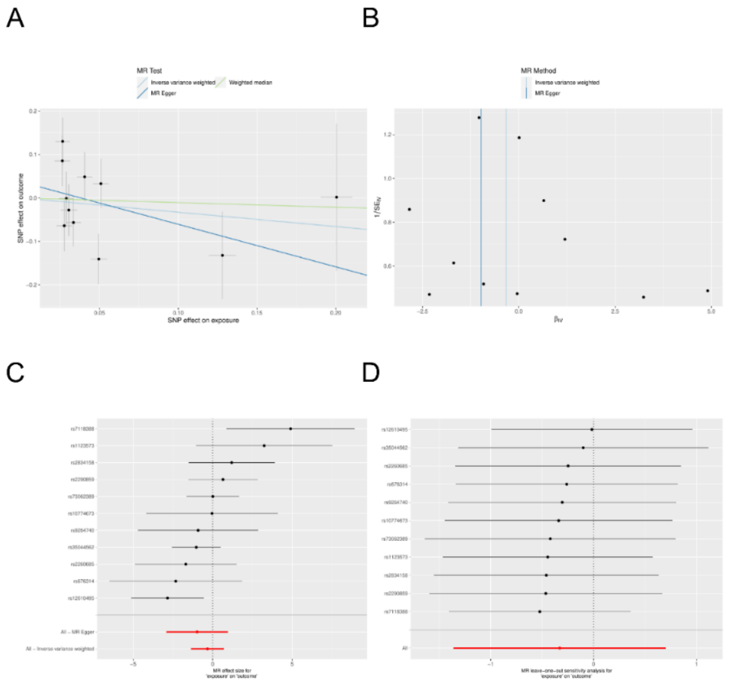

Figure.S17.1 Results of univariate Mendelian randomization of covid-19- Alopecia areata.
(A.scatter plots of causality, B. funnel plots, C.forest plots of each SNPs, D.leave-one-out plots)

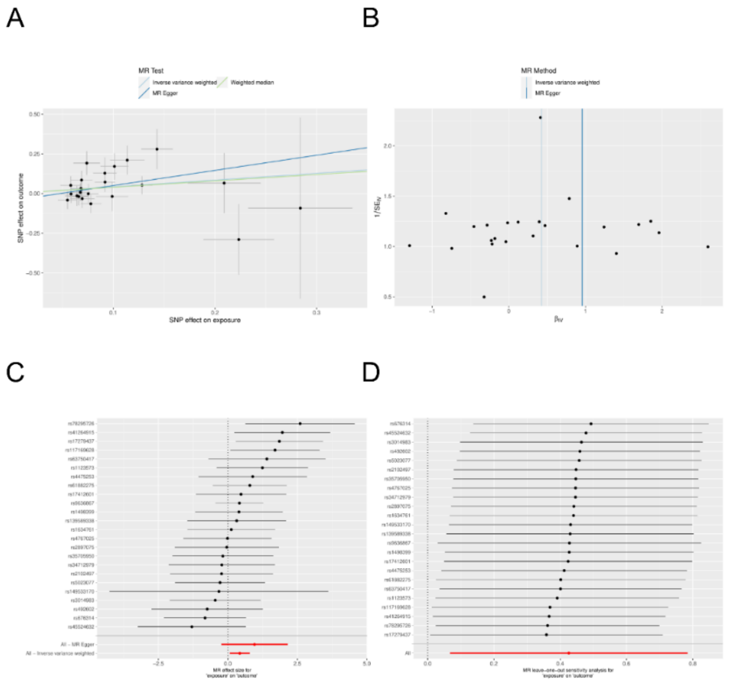

Figure.S17.2 Results of univariate Mendelian randomization of Hospitalized covid- Alopecia areata.
(A.scatter plots of causality, B. funnel plots, C.forest plots of each SNPs, D.leave-one-out plots)

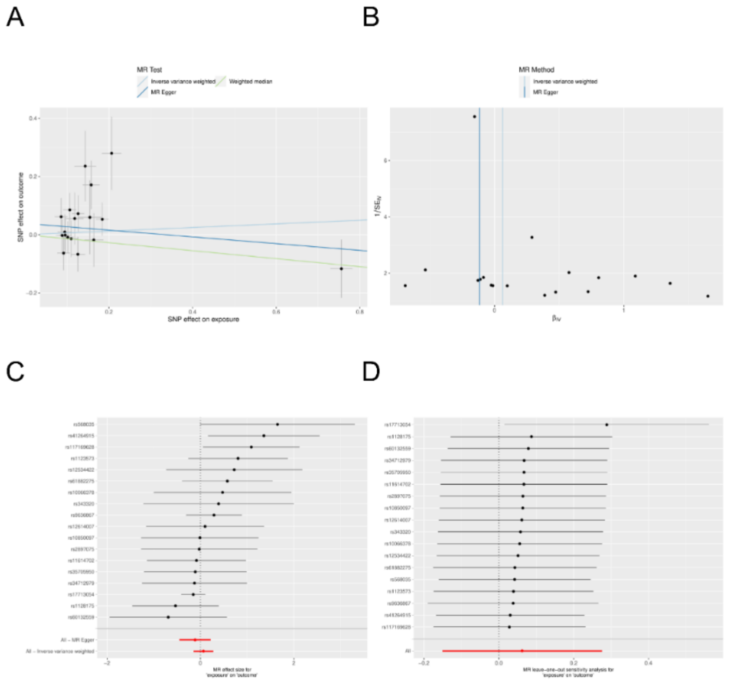

Figure.S17.3 Results of univariate Mendelian randomization of Very severe respiratory confirmed covid- Alopecia areata.

(A. scatter plots of causality, B. funnel plots, C.forest plots of each SNPs, D.leave-one-out plots）

FinnGen	UK Biobank	EBI	Meta-analysis		COVID-19
OR (95%CI)	OR (95%CI)	OR (95%CI)	OR (95%CI)	P value	post-COVID-19 syndrome
1.02 (0.72,1.46)	1.00 (1.00,1.00)	1.00 (1.00,1.00)	1.02 (0.72, 1.45)	0.91	Malaise and fatigue
	1.00 (0.98,1.46)				Shortness of breath walking on level ground
1.00 (0.84,1.20)	1.00 (1.00,1.00)	1.00 (1.00,1.00)	1.00 (0.84, 1.20)	1.00	Heart arrhythmia
0.72 (0.49,1.05)	1.00 (0.98,1.02)		0.90 (0.66, 1.22)	0.49	Eating disorders
1.50 (1.05,2.14)	1.00 (1.00,1.00)		1.50 (1.05, 2.14)	0.03	Myalgia
1.02 (0.91,1.14)	1.00 (1.00,1.01)	1.00 (1.00,1.00)	1.00 (1.00, 1.01)	0.99	Dorsalgia
0.90 (0.76,1.06)	1.00 (1.00,1.00)	1.00 (0.99,1.01)	1.00 (0.99, 1.01)	0.94	Joint pain
1.90 (1.04,3.50)	1.00 (1.00,1.00)	0.97 (0.90,1.03)	0.98 (0.91, 1.05)	0.52	Pain
0.99 (0.74,1.34)	1.00 (0.99,1.02)	1.07 (0.69 1.64)	1.00 (0.99, 1.02)	0.99	Sleeplessness / insomnia
0.81 (0.55,1.20)	0.96 (0.91,1.02)		0.96 (0.90, 1.01)	0.12	Memory loss
1.00 (0.99,1.02)	1.00 (0.99,1.00)	1.00 (0.99,1.00)	1.00 (1.00, 1.00)	1.00	Depression
1.05 (0.93,1.19)	1.00 (1.00,1.00)	0.96 (0.91,1.03)	0.98 (0.92, 1.03)	0.42	Anxiety
0.87 (0.71,1.08)	1.03 (0.92,1.15)		0.99 (0.90, 1.10)	0.88	Ever attempted suicide
0.95 (0.78,1.15)	1.00 (1.00,1.00)		0.95 (0.78, 1.15)	0.60	Alcohol use
1.68 (0.94,2.99)	1.00 (1.00,1.01)	1.13 (0.88,1.46)	1.39 (0.94, 2.04)	0.10	Tobacco use
1.08 (0.67,1.75)		0.84 (0.67,1.05)	0.88 (0.72, 1.08)	0.21	Erectile dysfunction
0.72 (0.26,2.02)					Alopecia areata

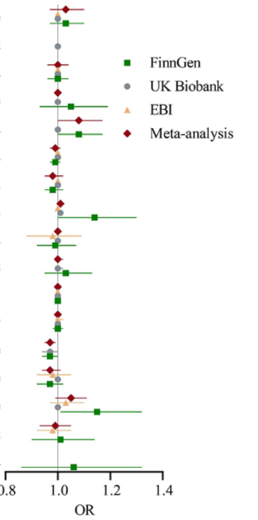

FinnGen	UK Biobank	EBI	Meta-analysis		Hospitalized COVID-19
OR (95%CI)	OR (95%CI)	OR (95%CI)	OR (95%CI)	P value	post-COVID-19 syndrome
0.97 (0.89,1.07)	1.00 (1.00,1.00)	1.00 (1.00,1.00)	0.97 (0.88, 1.06)	0.52	Malaise and fatigue
	1.00 (1.00,1.01)				Shortness of breath walking on level ground
1.00 (0.95,1.06)	1.00 (1.00,1.00)	1.00 (1.00,1.00)	1.00 (0.95, 1.06)	1.00	Heart arrhythmia
1.00 (0.86,1.15)	1.00 (0.99,1.01)		1.00 (0.99, 1.01)	1.00	Eating disorders
1.04 (0.92,1.17)	1.00 (1.00,1.00)		1.04 (0.92, 1.17)	0.52	Myalgia
0.98 (0.95,1.01)	1.00 (1.00,1.00)	1.00 (1.00,1.00)	0.98 (0.95, 1.01)	0.20	Dorsalgia
0.99 (0.95,1.03)	1.00 (1.00,1.00)	1.00 (1.00,1.01)	1.00 (0.99, 1.00)	0.95	Joint pain
1.15 (0.97,1.37)	1.00 (1.00,1.00)	1.00 (1.00,1.00)	1.00 (1.00, 1.01)	0.96	Pain
0.95 (0.86,1.05)	1.00 (0.99,1.01)	0.96 (0.83,1.11)	1.00 (0.99, 1.01)	0.92	Sleeplessness / insomnia
1.01 (0.88,1.16)	1.00 (0.98,1.02)		1.00 (0.98, 1.02)	0.98	Memory loss
1.00 (0.99,1.00)	1.00 (1.00,1.00)	1.00 (1.00,1.00)	1.00 (1.00, 1.01)	1.00	Depression
1.00 (0.96,1.04)	1.00 (1.00,1.00)	1.00 (0.98,1.03)	1.00 (0.98, 1.02)	1.00	Anxiety
0.98 (0.94,1.02)	0.98 (0.94,1.03)		0.98 (0.95, 1.01)	0.19	Ever attempted suicide
0.95 (0.88,1.02)	1.00 (1.00,1.00)		0.95 (0.88, 1.02)	0.17	Alcohol use
1.18 (0.98,1.42)	1.00 (1.00,1.00)	1.00 (0.92,1.09)	1.03 (0.95, 1.11)	0.47	Tobacco use
0.99 (0.83,1.17)		1.15 (1.03,1.29)	1.10 (1.00, 1.21)	0.05	Erectile dysfunction
1.53 (1.07,2.19)					Alopecia areata

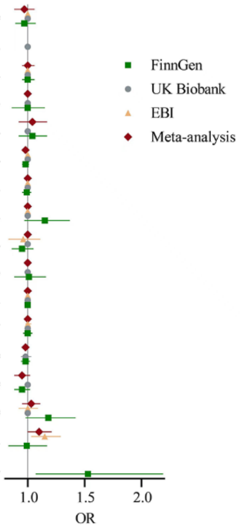

FinnGen	UK Biobank	EBI	Meta-analysis		Severe COVID-19
OR (95%CI)	OR (95%CI)	OR (95%CI)	OR (95%CI)	P value	post-COVID-19 syndrome
1.03 (0.97,1.10)	1.00 (1.00,1.00)	1.00 (1.00,1.00)	1.03 (0.97, 1.10)	0.36	Malaise and fatigue
	1.00 (1.00,1.01)				Shortness of breath walking on level ground
1.00 (0.96,1.04)	1.00 (1.00,1.00)	1.00 (1.00,1.00)	1.00 (0.96, 1.04)	1.00	Heart arrhythmia
1.05 (0.93,1.19)	1.00 (1.00,1.01)		1.00 (1.00, 1.01)	0.98	Eating disorders
1.08 (1.00,1.17)	1.00 (1.00,1.00)		1.08 (1.00, 1.17)	0.05	Myalgia
0.99 (0.97,1.01)	1.00 (1.00,1.00)	1.00 (1.00,1.00)	0.99 (0.97, 1.01)	0.33	Dorsalgia
0.98 (0.95,1.02)	1.00 (1.00,1.00)	1.00 (1.00,1.00)	0.98 (0.95, 1.02)	0.27	Joint pain
1.14 (1.00,1.30)	1.01 (1.00,1.01)	1.00 (1.00,1.00)	1.01 (1.01, 1.02)	0.00	Pain
0.99 (0.92,1.07)	1.00 (1.00,1.01)	0.98 (0.88,1.09)	1.00 (1.00, 1.00)	0.97	Sleeplessness / insomnia
1.03 (0.95,1.13)	1.00 (0.99,1.02)		1.00 (0.99, 1.02)	0.91	Memory loss
1.00 (0.99,1.00)	1.00 (1.00,1.00)	1.00 (1.00,1.00)	1.00 (1.00, 1.01)	1.00	Depression
1.00 (0.98,1.02)	1.00 (1.00,1.00)	1.00 (0.99,1.02)	1.00 (0.99, 1.01)	1.00	Anxiety disorders
0.97 (0.94,1.00)	0.97 (0.94,1.00)		0.97 (0.95, 0.99)	0.01	Ever attempted suicide
0.970 (0.92,1.02)	1.00 (1.00,1.00)	0.98 (0.92,1.05)	0.97 (0.94, 1.01)	0.20	Alcohol use
1.15 (1.01,1.32)	1.00 (1.00,1.00)	1.03 (0.97,1.10)	1.05 (0.99, 1.11)	0.08	Tobacco use
1.01 (0.90.1.14)		0.98 (0.92,1.05)	0.99 (0.93, 1.05)	0.66	Eerectile dysfunction
1.06 (0.86,1.32)					Alopecia areata

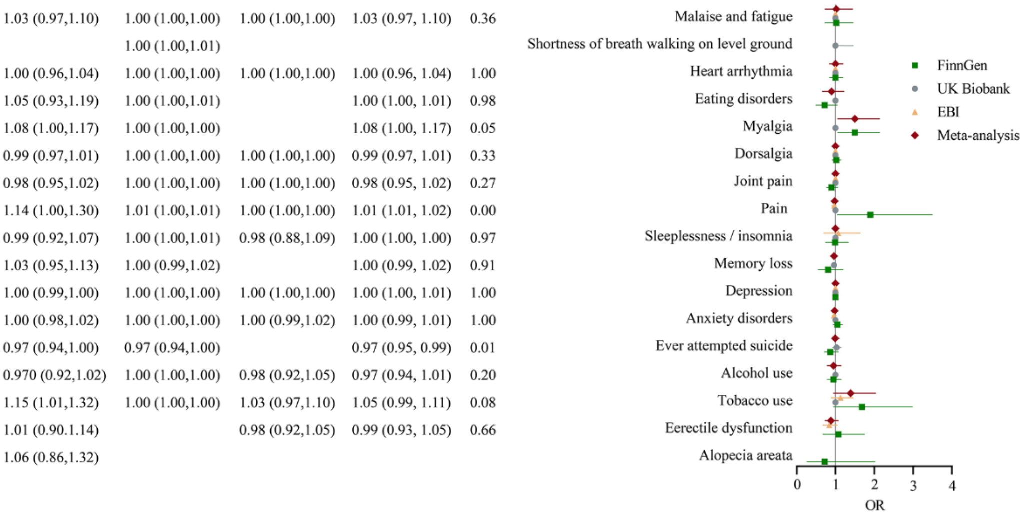

Figure.S18. Results of Mendelian randomization analysis and meta-analysis using Finngen, UK biobank and EBI databases as outcome factors.

EBI, European Bioinformatics Institute

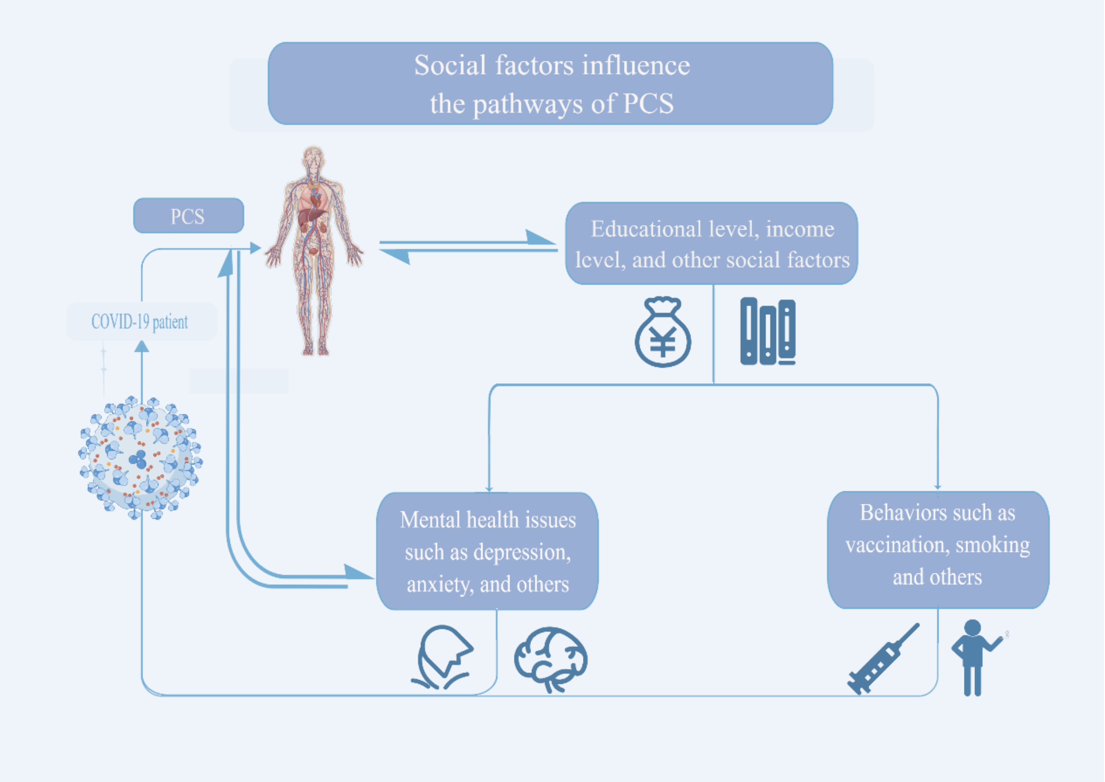

Figure.S19. The influence of educational attainment, socioeconomic status, and various othe r societal factors on the effects of PCS.

PCS,post-covid-19 syndrome
